# Epidemiological Data and Antimicrobial Resistance of *Campylobacter* spp. in Portugal from 13 Years of Surveillance

**DOI:** 10.3390/pathogens13020147

**Published:** 2024-02-06

**Authors:** Andreia Duarte, Luísa Pereira, Maria-Leonor Lemos, Miguel Pinto, João Carlos Rodrigues, Rui Matias, Andrea Santos, PTCampyNet, Mónica Oleastro

**Affiliations:** 1 Chemistry Department, Sciences Faculty, University of Beira Interior, Rua Marquês d’Ávila e Bolama, 6201-001 Covilhã, Portugal; andreia.duarte@ubi.pt; 2 CMA-UBI, Centre of Mathematics and Applications, University of Beira Interior, Rua Marquês d’Ávila e Bolama, 6201-001 Covilhã, Portugal; lpereira@ubi.pt; 3 Infectious Diseases Department, National Institute of Health Doutor Ricardo Jorge (INSA), 1649-016 Lisbon, Portugal; m.leonor.lemos@insa.min-saude.pt (M.-L.L.); joao.rodrigues@insa.min-saude.pt (J.C.R.); rui.matias@insa.min-saude.pt (R.M.); andrea.santos@insa.min-saude.pt (A.S.); 4 ICBAS-Institute of Biomedical Sciences Abel Salazar, University of Porto, 4050-313 Porto, Portugal; 5 Genomics and Bioinformatis Unit, National Institute of Health Doutor Ricardo Jorge (INSA), 1649-016 Lisbon, Portugal; miguel.pinto@insa.min-saude.pt

**Keywords:** *Campylobacter* infection, epidemiology, surveillance, notification, antibiotic resistance, WGS, resistance genetic determinants

## Abstract

This study extensively analyzed campylobacteriosis surveillance in Portugal from 2009 to 2021, aiming to investigate demographic shifts, seasonal variations, and antimicrobial resistance (AMR) within *Campylobacter* isolates. Surveillance network and sentinel laboratory-based system data revealed a substantial under-notification of campylobacteriosis cases, suggesting an underestimated disease burden. Notification rates exhibited a paradigm shift, with a notable prevalence among the pediatric population, particularly in children aged 1–4 years, diverging from European reports. Additionally, an emerging trend of *Campylobacter* infections in younger adults (15–44 years) was observed. The study unveiled a unique seasonal distribution of cases, defying typical summer peaks seen elsewhere. AMR analysis revealed high resistance to ciprofloxacin and tetracycline, in both *C. jejuni* (93.7% and 79.2%, respectively) and *C. coli* (96.5% and 93.2%, respectively), stable throughout the studied period (2013–2021). *C. coli* exhibited significantly higher resistance to erythromycin, gentamicin, ampicillin and ertapenem compared to *C. jejuni* (*p* < 0.001). Multilocus Sequence Typing (MLST) data demonstrated the distribution of resistance markers across diverse sequence types, challenging the notion of a clonal origin for multidrug-resistant isolates. In conclusion, the study highlights the need for enhanced surveillance and raises concerns about alarming AMR levels, recommending the implementation of whole-genome sequencing (WGS)-based surveillance for a deeper comprehension of disease patterns and an evolving AMR landscape.

## 1. Introduction

*Campylobacter* spp. is the most common etiological agent of bacterial gastroenteritis in Europe, with *Campylobacter jejuni* as the main cause of campylobacteriosis, followed by *Campylobacter coli*. *Campylobacter* is a commensal microorganism of the gastrointestinal tract of many wild and domestic animals that serve as reservoirs for transmission. Most of the infections manifest as sporadic cases with unknown sources, often arising from the consumption of contaminated food or water, the mishandling of contaminated food, interaction with domestic and farm animals, or exposure to contaminated environments [1].

*Campylobacter* infection typically manifests with symptoms ranging from mild watery diarrhea to severe inflammatory bloody diarrhea, accompanied by abdominal pain, headache, nausea, and fever. More rarely, the infection can result in autoimmune complications such as Guillain–Barré and Miller Fisher Syndromes. The onset of symptoms generally occurs between two and five days post-infection, with a duration of up to ten days. The majority of cases of enteric campylobacteriosis follow a self-limiting course, thereby infrequently necessitating antimicrobial intervention [2]. However, in cases where the condition worsens, particularly in immunocompromised individuals, antibiotic therapy may become imperative, thereby highlighting the increasing threat of antibiotic-resistant *Campylobacter* strains [2,3]. The surveillance of such resistance stands as a pivotal parameter in combatting campylobacteriosis.

In Europe, the surveillance of campylobacteriosis is under the oversight of the European Center for Disease Prevention and Control (ECDC). This entity undertakes the coordination, analysis, and dissemination of surveillance data collected from countries within the European Union (EU) and the European Economic Area (EEA) [4]. However, since the data are obtained individually at the national level, the accuracy of the analyses depends largely on the effectiveness and implementation of programs within each respective country [5].

In the context of Portugal, limited published data concerning the epidemiology of campylobacteriosis exist, and the only comprehensive study addressing the epidemiological aspects of human infection dates back to 1992 [6]. Subsequent investigations have been limited in scope and with reduced datasets, primarily focusing on the genotypic and phenotypic attributes, particularly those associated with antimicrobial susceptibility of *Campylobacter* isolates sourced from animals, food, or humans [7,8,9,10,11,12].

The present study aims to bridge the information gap by presenting up-to-date epidemiological data on *Campylobacter* spp. infection in Portugal. Employing a sentinel laboratory-based surveillance approach, the study covers the nation’s three most densely populated regions, from January 2009 to December 2021. The primary objective is to provide a current overview of campylobacteriosis in Portugal, delineate temporal trends, estimate disease burden, and monitor antimicrobial-resistant infections.

## 2. Materials and Methods

### 2.1. Study Design

This is a retrospective study carried out in *Campylobacter* spp. isolates from patients with diarrhea and/or other gastrointestinal symptomatology, collected from different Portuguese hospitals, from January 2009 to December 2021. The epidemiological, sociodemographic, microbiological, and clinical data were collected for positive *C. jejuni* and *C. coli* cases, from the clinical laboratories and from the National Reference Laboratory (NRL), at INSA. As part of the routine surveillance, data from patients’ medical records were provided from the primary laboratories (clinical specimen, age and gender, symptoms, time (days) from onset of symptoms to initial medical visit, and epidemiological information) while microbiological data (species identification/confirmation and antimicrobial susceptibility) were collected from the NRL reports. The data analysis, including the statistical analyses, was performed after the data collection phase.

At the NRL, species identification was confirmed or determined by a real-time fluorescence resonance energy transfer PCR, specific for *C. jejuni* and *C. coli* [13], targeting the *gyrA* gene, or by MALDI-TOF (VITEK^®^ MS, bioMérieux, Marcy l’Etoile, France). Antimicrobial susceptibility testing (AST) was implemented in the processing of samples in 2013. Antimicrobial resistance (AMR) against the four priority antimicrobials (ciprofloxacin (CIP), erythromycin (ERY), tetracycline (TCY), and gentamicin (GEN) (cut-offs of EUCAST [14,15])) was analyzed, as well as against the optional antibiotics (ampicillin (AMP), amoxicillin–clavulanic acid (AMC)) by disk diffusion, and for ertapenem (ETP) by E-test (cut-offs of the Comité de l’antibiogramme de la Société Française de Microbiologie [16]).

From 2016 up to 2021, subsets of isolates were subjected to NexteraXT library preparation (Illumina, San Diego, CA, USA) prior to paired-end sequencing on an Illumina instrument (Illumina), according to the manufacturer’s instructions. Genome sequences were assembled using the INNUca v4.2.2 pipeline (https://github.com/B-UMMI/INNUca) (accessed on 18 December 2023), an integrative bioinformatics pipeline for read quality analysis and de novo genome assembly [17]. Whole-genome sequencing (WGS) data were used to perform Multilocus Sequence Typing, via the PubMLST online platform [18], and to screen for the presence of AMR-associated genetic markers, using Resfinder v4.3.2 [19]. In order to infer the phylogenetic relationship between isolates the MLST data were used.

All assembled genomes used in the present study were deposited in the European Nucleotide Archive (ENA), whereby the accession numbers are described in Table S7.

### 2.2. Statistical Analyses

Categorical variables were presented with their relative and absolute values while quantitative ones were expressed as mean and standard deviation, or median and interquartile range. Regarding patients’ age, and depending on the analysis, patients were either defined as pediatric (age ≤ 15 years) or adult population, or stratified in seven age groups (<1, 1–4, 5–14, 15–24, 25–44, 45–64, and 65+ years). For statistical analysis, Student’s *t*-test, Mann–Whitney, chi-squared, and Fisher’s exact tests were applied using the statistical software package SPSS 28.0 for Windows (SPSS, Chicago, IL, USA). A binary logistic regression analysis was performed to determine independent risk factors for resistance to different antibiotics. *p* < 0.05 indicates a statistically significant test result.

## 3. Results

### 3.1. Data from the Surveillance Network

Campylobacteriosis has been a notifiable disease in Portugal since 2015. Cases are reported by clinicians in an electronic platform called SINAVE (stands for National Epidemiological Surveillance System), and since 2017, laboratory notification using the same platform has also been mandatory. In parallel, a sentinel laboratory-based surveillance of this disease has been in place since 2009, whose networks comprise primary laboratories mainly from hospitals, both public and private, which operate on a voluntary basis. These laboratories send all *Campylobacter* spp. isolates, collected throughout the year, to the NRL located at the National Institute of Health Doutor Ricardo Jorge. For each isolate, anonymized epidemiological, demographic, microbiological, and clinical data are also requested. Currently, the sentinel laboratory network comprises nine public hospital centers and three public hospitals from the national health system, from the three most populated geographical areas of Portugal, representing three among five regions from mainland Portugal, from the second tier of Nomenclature of Territorial Units for Statistics level 2 (NUTSII): North (six hospital centers, one hospital), Center (one hospital center), and Metropolitan Lisbon Area (two hospitals centers, two hospitals), with a total catchment population of ~9,300,000. In addition, two private networks of clinical analysis and medical diagnosis laboratories, both covering private primary laboratories at the hospital and community levels operating in mainland Portugal, were also included.

The NRL’s inability to keep pace in terms of sample processing with the exponentially increased number of isolates received, forced, as of 2019, the random selection of around 40% of samples for species confirmation/determination and AST.

### 3.2. Temporal Distribution of Cases of Campylobacter Infection Received from 2009 to 2021

Accompanying the expansion of the laboratory network, the number of isolates sent to the NRL has been increasing over time, with a mean number of 263 isolates, from 2009 to 2012, ≈465 isolates from 2013 to 2016, and 751 from 2017 to 2021, with its highest number reached in 2019, with a total of 916 isolates received (Table 1). Simultaneously, the number of laboratory-notified cases of *Campylobacter* infections at a national level, in SINAVE, increased as well, from 596 to 973 cases in the period 2017–2021 [1]. However, neither the number of annual *Campylobacter* infections nor their increasing trend can be accurately estimated with the available data, as the number of isolates received in the NRL is higher than the cases notified in SINAVE (except in 2021), and around 50% of those are not notified. Indeed, notification to SINAVE is no better for the laboratories participating in sentinel surveillance, with a relevant heterogeneity in the notification rates per 100,000 population among the three regions: 9.1, 7.1, and 3.9 for North, Center, and Metropolitan Lisbon Area, respectively.

### 3.3. Seasonal Variation in the Number of Campylobacter Infection Cases

**Figure 1:**
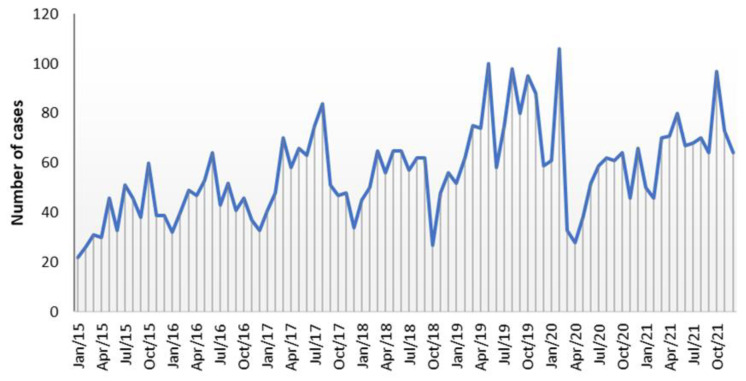
Distribution of the number of cases of *Campylobacter* infections according to the month of sampling, considering the total of cases received in the NRL in the most representative period spanning from 2015 to 2021.

The graph of the number of cases received per month does not show a clear seasonal regularity over the years (Figure 1). Despite this, a slight seasonal recrudescence of cases of *Campylobacter* infections was noted during the mid-spring/summer period, with May and August as the months with the highest cumulative number of *Campylobacter* cases. With the opposite trend, a small decay in the monthly prevalence of *Campylobacter* infections was observed in the winter months, with January and December reporting the lowest numbers of cumulative cases of infection. Outside the mid-spring/summer period, three peaks of infection, associated mainly with *C. jejuni*, were observed occurring in October 2019 (95 cases), October 2021 (97 cases), and in February 2020 (106 cases), corresponding to 1.6 and 2.0 times more cases than the mean number for those months (62 and 54, respectively) (Figure 1).

### 3.4. Demographic and Clinical Data

In the following analysis, the 5205 *Campylobacter* spp. isolates from human infections (one per patient, not travel-related) processed by the NRL in the period spanning from 2009 to 2021 were considered, being 4590 *C. jejuni* and 615 *C. coli*. Most of the cases reported the isolation of *Campylobacter* spp. from stool samples (5157, 99.08%), followed by blood samples (46, 0.88%). The isolation of *C. jejuni* from a gastric biopsy and of *C. coli* from a bile sample was also reported.

Overall, during the studied period, *C. jejuni* accounted for 83.9% to 92.3% of *Campylobacter* infections and was significantly more frequent than *C. coli*, which represented 7.7% to 16.1% of the infections. In the period 2009–2016, a progressive but not significant increase in the number of *C. jejuni* was noted from year to year. In contrast, in the time period 2017–2020, there was a decrease in the number of *C. jejuni* isolates (Table 2). The number of *C. coli* infections remained relatively stable during the study period, although showing a few fluctuations (Table 2).

The patients’ mean age was 12.79 years (*n* = 5120) (ranging from 1 day to 96 years). The majority of the cases (77.7%; 3979/5120) were from the pediatric population and 60.2% of the patients were of male gender (3096/5144).

**Figure 2:**
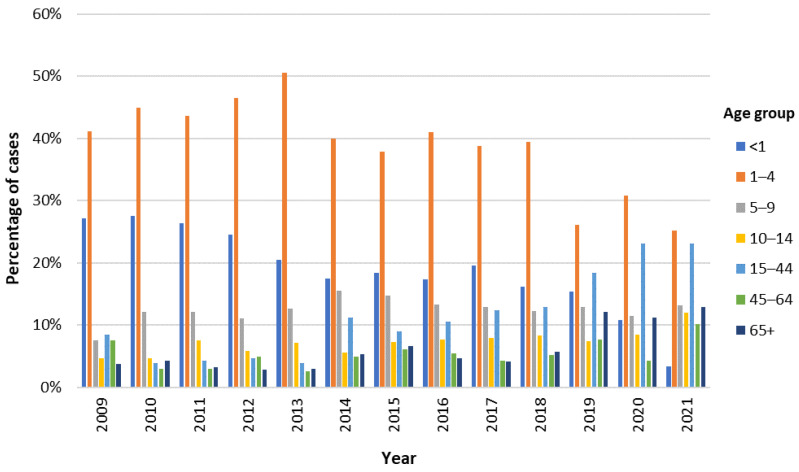
Percentage of cases of *Campylobacter* infections by age group (in years) in the time period spanning from 2009 to 2021.

Considering the temporal distribution of cases in each age group, children from 1 to 4 years old represented the group with the highest percentage of cases every year (Figure 2). After reaching its highest value in 2013 (50.5%), this percentage has been decreasing, only accounting for 25.2% of the cases in 2021. In contrast, the percentage of cases in the adult population has been increasing, with a particular notability in the group of younger adults (15–44 years), which in 2020 and 2021 accounted for 23.1% of *Campylobacter* infections each (Figure 2).

The main reported symptom was diarrhea, including bloody diarrhea, affecting 3405 out of 3673 (92.7%) of the patients. Other reported symptoms included abdominal pain, fever, and vomiting, to a lesser extent (Table 3).

Bloody diarrhea was significantly more likely in the pediatric than in the adult population (OR = 4.7367; 95% CI: 0.5399–0.8368), while abdominal pain (OR = 0.5018; 95% CI: 0.4038–0.6236) and fever (OR = 0.6721; 95% CI: 0.5399–0.8368) were less likely to occur in the pediatric than in the adult population (Table 4).

The distribution of the main symptoms according to the seven defined age groups corroborates the former associations, showing that bloody diarrhea was significantly associated with younger children (≤4 years), while in contrast, it is a rare condition in patients aged 65+ years (Table S1).

In addition to the main symptoms associated with *Campylobacter* infection, several patients described underlying medical conditions: 43 patients presented immunosuppression, 37 had colitis, and 15 showed symptoms of infection other than *Campylobacter*. Concerning extra-gastrointestinal conditions that can be associated with the infection, three patients reported the neurologic Guillain–Barré/Miller Fisher syndromes. Patients with immunosuppression were significantly more prone to develop bacteremia (positive blood culture) (58.1% vs. 41.9%; *p* = 0.03).

### 3.5. Distribution of Cases of Campylobacter Infection According to Age, Gender, and Geographic Region

To evaluate if the prevalence of *Campylobacter* infection was associated with age, gender, and geographic region of sample provenance, a comparison of the distribution of positive samples according to these three criteria was performed (Table 5). The association between campylobacteriosis and gender was statistically significant, with 3096 male (60.2%, 95% CI: 58.8–61.5) and 2048 female (39.8%, 95% CI: 38.5–41.2) patients reported as positive for *Campylobacter* spp. isolation. Opposed to this trend, the risk of infection was significantly lower in males than in females for the age group 15–44 years old (RR 0.799; 95%CI 0.684–0.933; *p* = 0.0045), while for the remaining age groups, the difference was not statistically significant (Table S2).

When considering the distribution of *Campylobacter* infection cases by age groups (Table 5), the highest rate was observed in young children, with 2001 cases (39.1%) in 1–4-year-old infants, followed by children aged < 1 year (934, 18.2%). By species, the highest number of cases of *C. jejuni* infection was observed for the age groups from 1 to 4 years (40.0%) and 0 to 1 years (18.4%), with the same observed for *C. coli* infections, 1–4 years (32.1%) followed by the age group 0–1 years (17.0%). However, considering the distribution of species per age group, it is shown that the prevalence of *C. jejuni* infection is significantly higher than that of *C. coli* in children aged 1–4 years old, while from the age of 15 years, the burden of *C. coli* infection is significantly higher (*p* < 0.05) (Table 5). The distribution of *Campylobacter* species for pediatric and adult populations per year also corroborates this trend, with the rate of *C. coli* being around 2-fold higher in adults than in children, except in the year 2013 (Table S3).

The distribution of cases according to the hospital’s geographical origin showed higher levels in the north of the country (44.7%) than in Metropolitan Lisbon Area (35.1%) and in the central region (15.2%) (Table 5). However, these numbers are influenced by the number and size of the participating hospitals, affecting the number of samples submitted to the NRL.

### 3.6. Other Epidemiological Data

Of all the considered cases, only 40 (0.768%) reported a possible transmission route: 42.5% indicated the consumption of untreated water from private drinking water wells, 17.5% a possible foodborne association, and 22.5% reported a traveling history soon before the infection. This contrasts with the notified data from SINAVE, retrieved from the epidemiological surveys, for which food was the main vehicle reported (68.7%), followed by person-to-person (19.0%), water (8.0%), and animal transmission (4.3%). According to records from the primary laboratories, only a small proportion of cases reported an association with outbreaks (43 of 5203 cases).

The median (interquartile range) time elapsed from the onset of symptoms to the initial medical visit was three days (IQR: 2–5 days), but it could be extended beyond 16 days. The median delay was shorter for men [3 (IQR: 2–5) days] than for women [4 (IQR: 2–6) days] (*p* < 0.05). The time interval from the first symptoms to the initial medical visit was not significantly different in the seven age groups considered (*p* = 0.896) (Table S4).

### 3.7. Antimicrobial Resistance of Campylobacter spp. Strains

Antimicrobial susceptibility was determined for 2174 *Campylobacter* spp. isolates, 1807 (83.1%) *C. jejuni* and 367 (16.9%) *C. coli*, and the results are summarized in Table 6. The *Campylobacter* spp. isolates were analyzed for AMR against seven antibiotics, whether priority or optional for treatment of campylobacteriosis: ciprofloxacin, erythromycin, tetracycline, and gentamicin from 2013 to 2021, ampicillin and ertapenem, from 2017 to 2021, and ampicillin from 2018 to 2021.

**Figure 3:**
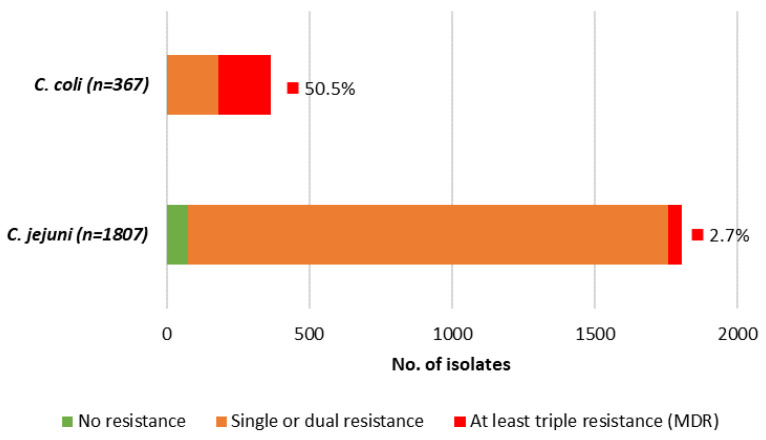
Proportion of *Campylobacter* spp. isolates that are multidrug resistant (MDR), resistant to one and/or two antimicrobials and completely susceptible, while considering the four priority antimicrobials (CIP—ciprofloxacin, ERY—erythromycin, TET—tetracycline, GEN—gentamicin).

Overall, both species presented extremely high (>93%) level of resistance to ciprofloxacin, while for tetracycline, *C. jejuni* presented very high level of resistance (≥80%), and *C. coli* extremely high level (>93%). For both antibiotics, resistance was significantly higher for *C. coli* than for *C. jejuni* (*p* = 0.043 for ciprofloxacin and *p* < 0.001 for tetracycline) (Table 6). Regarding erythromycin, *C. coli* presented high level of resistance (52.3%) compared to the low level determined for *C. jejuni* (3.3%) (*p* < 0.001). The level of resistance to gentamicin was overall low; however, the difference was also significant when comparing *C. coli* (2.5%) with *C. jejuni* (0.1%) (*p* < 0.001) (Table 6). Generally, a very low level of circulating *Campylobacter* strains was fully susceptible (<2% in *C. coli*, ~4% in *C. jejuni*), while multidrug resistance (MDR)—here defined as combined resistance to ciprofloxacin, erythromycin, and tetracycline—was frequently found in *C. coli* (50.5%) and was rare in *C. jejuni* (2.7%) (Figure 3).

**Figure 4:**
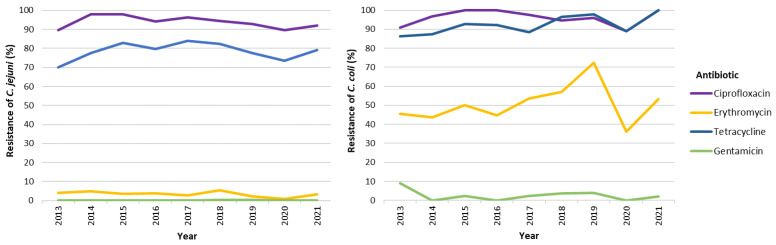
Evolution of the resistance rate for *Campylobacter jejuni* (at the **left**) and *Campylobacter coli* (at the **right**) to the four priority antimicrobials (CIP, ERY, TET, GEN), from 2013 to 2021.

The evolution of resistance to antimicrobial agents from 2013 to 2021 is summarized in Figure 4 and Table S5 and shows that, for both species, the level of resistance to the four priority antimicrobials has remained relatively constant over the studied years, the only exception being the decrease in resistance to erythromycin for *C. coli* in 2020 (36.1%), which returned to the higher rates in 2021 (Figure 4 and Table S5). Regarding the optional antibiotics tested, most of the strains were resistant to ampicillin (>75%), whereby this trend was stable in the period covered, i.e., 2018–2021. In contrast, for ertapenem, and considering the minimal inhibitory concentration (MIC) breakpoint > 1 mg/L, a very low-to-low proportion of strains presented resistance, although again, significantly higher in *C. coli* than in *C. jejuni* (6.5% vs. 0.7%, *p* < 0.001) (Table 6). The MIC variation for susceptible isolates was 0.016–0.75 mg/L, while for non-susceptible isolates, it ranged from 2.0 to >32 mg/L for *C. coli* and 1.5 to 4 mg/L for *C. jejuni*. Regarding amoxicillin–clavulanic acid, a very low-to-low proportion of the strains (0.2% in *C. jejuni*, 3.9% in *C. coli*; *p* < 0.001) showed decreased susceptibility, with inhibition zone diameters between 14 and 19 mm, corresponding to MICs varying between 4 and 8 mg/L. This decreased susceptibility was only perceived in the years 2017 and 2018 (Table S5).

Demographic features and species associated with resistance were explored through a univariate logistic regression (Table S6), showing that resistance to ciprofloxacin, tetracycline, and ampicillin was significantly less likely to occur in the age groups 15–44 and 45+ compared to the pediatric population. As previously observed, *C. coli* strains were significantly more likely to have resistance to erythromycin and amoxicillin–clavulanic acid compared to *C. jejuni* strains. Compared to the Metropolitan Lisbon Area, ertapenem resistance was less likely to occur in the central and northern regions (Table S6).

Variables from the univariate analysis (Table S5) were subsequently included in a multivariate analysis (Table 7) confirming that age significantly decreases the odds of resistance to ampicillin, ciprofloxacin, and tetracycline, and that *C. coli* strains are significantly more likely than *C. jejuni* strains to resist all tested antibiotics, except ciprofloxacin and ampicillin. Accordingly, *C. coli* isolates had significantly minor inhibition diameter zones than *C. jejuni*, except for ampicillin.

### 3.8. Campylobacter spp. Typing and AMR-Associated Genetic Markers

In order to gain knowledge regarding the molecular epidemiology of circulating *Campylobacter* isolates in Portugal and to assess the correlation between phenotypic resistance and the presence of resistance genes and/or point mutations, a total of 380 *Campylobacter* spp. isolates (136 *C. coli* and 244 *C. jejuni*), collected between 2016 and 2021, were randomly selected and subjected to molecular typing (Table 1).

**Figure 5:**
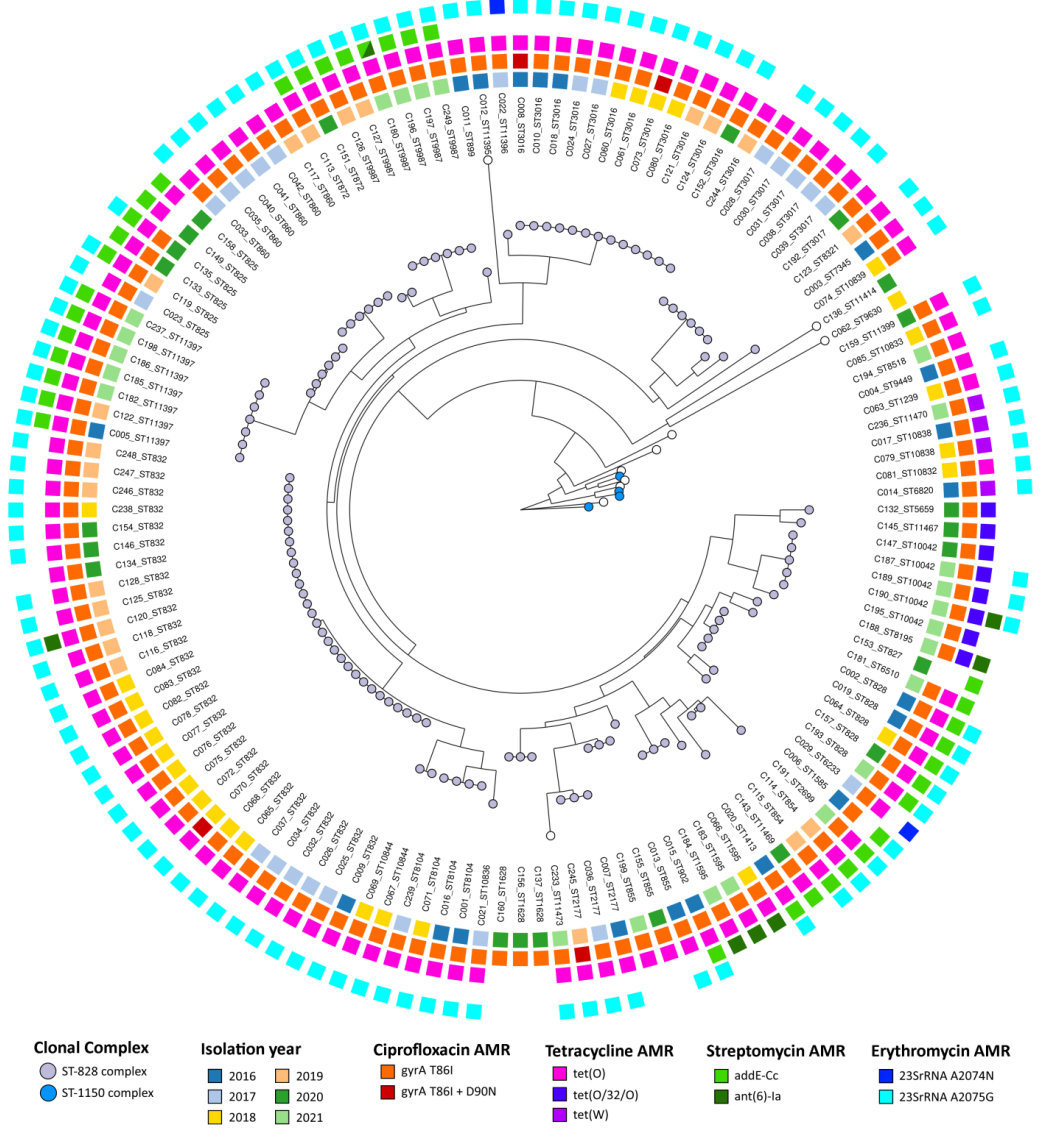
Multilocus Sequence Typing-based phylogenetic tree of 136 *Camplylobacter coli* isolates from 2016 to 2021. Neighbor-joining tree was reconstructed based on MLST profile data using GrapeTree [20]. Each node, corresponding to an isolate, is colored according to MLST clonal complex. Sequence type (ST) is indicated next to the isolate’s ID. Metadata blocks, from inner to outer, display (i) isolation year and presence of AMR-associated genetic markers for (ii) ciprofloxacin, (iii) tetracycline, (iv) streptomycin, and (v) erythromycin.

MLST-based genetic diversity analysis of *C. coli* (Figure 5) showed that most isolates belong to clonal complex ST-828 (89.7%, 122/136), composed of 34 distinct STs, followed by ST-1150 complex (2.9%, 4/136), composed of 3 distinct STs, while for 10 isolates, no clonal complex could be assigned (Figure 5 and Table S7). Regarding AMR-associated markers, 96.3% (127/136) of isolates carried the *gyrA* T86I amino acid alteration associated with ciprofloxacin resistance, with four of these also carrying the D90N alteration. Tetracycline plasmid-encoded resistance was observed in 94.9% through the presence of either the *tet(O)*, *tet(O/32/O)* or *tet(W)* variants (Figure 5). Two genetic markers associated with streptomycin resistance were detected in 29.4% (40/136) of *C. coli* isolates, with 34 isolates carrying the *aadE-Cc* genes, six isolates carrying the *ant(6)-Ia* genes, and one isolate displaying both. For erythromycin, 81.6% (111/136) of all isolates harbor a resistance-associated mutation in the *23S rRNA* gene (i.e., A2075G or A2074N) (Figure 5 and Table S7). There was a perfect correlation between resistance phenotype and genetic resistance markers for the tested antibiotics ciprofloxacin, erythromycin, and tetracycline. The resistance markers were distributed among the different STs circulating over the studied time period, challenging the notion of a clonal origin for MDR isolates.

**Figure 6:**
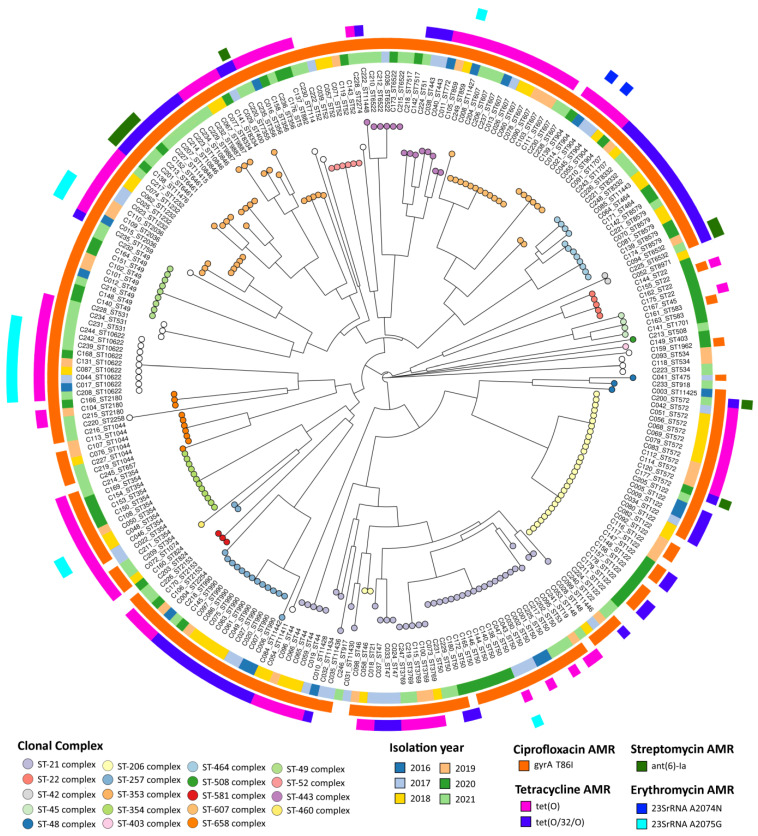
Multilocus Sequence Typing-based phylogenetic tree of 244 *Camplylobacter jejuni* isolates from 2016 to 2021. Neighbor-joining tree was reconstructed based on MLST profile data using GrapeTree [20]. Each node, corresponding to an isolate, is colored according to MLST clonal complex. Sequence type (ST) is indicated next to the isolate’s ID. Metadata blocks, from inner to outer, display (i) isolation year and presence of AMR-associated genetic markers for (ii) ciprofloxacin, (iii) tetracycline, (iv) streptomycin, and (v) erythromycin.

Regarding *C. jejuni* isolates (Figure 6), a higher genetic diversity was observed, with isolates belonging to at least 19 defined clonal complexes, totaling 77 different STs among the 244 isolates, distributed across the analyzed time period. The most prevalent clonal complex detected was the ST-21 complex (16.8%, 41/244), which enrolled 14 different STs and was consistently observed in each year of the study period (Table S7). Most isolates (89.3%, 218/244) carried the *gyrA* T86I ciprofloxacin resistance-associated alteration, while only 3.7% (9/244) carried the *ant(6)-Ia* gene associated with streptomycin resistance (Figure 6). Additionally, 67.6% (165/244) of *C. jejuni* isolates presented either the *tet(O)* or *tet(O/32/O)* tetracycline resistance-associated genes, while *tet(W)* was not present. Contrasting with *C. coli*, only 6.6% of the typed isolates (16/244) harbored the well-known erythromycin resistance-associated mutation A2075G in the *23S rRNA* gene, in addition to two isolates with the mutation in position 2074, corroborating the phenotypic data. The 18 erythromycin resistant isolates were distributed across seven different STs, although the ST-10662 was overrepresented, accounting for half of the resistant isolates and being present throughout the studied period (2016–2021) (Figure 5 and Table S7). Similar to *C. coli*, a perfect correlation between resistance phenotype and genetic resistance markers for the tested antibiotics ciprofloxacin, erythromycin, and tetracycline was observed for *C. jejuni*.

Genomic data (MLST type and antimicrobial resistance genetic marker) of the *Campylobacter* spp. isolates enrolled in the present study are described in Table S7.

## 4. Discussion

This study examined the laboratory surveillance data on campylobacteriosis in Portugal for a 13-year period from January 2009 to December 2021. The findings shed light on several key aspects of this disease, including notification rates, demographic trends, seasonal variation, and AMR patterns.

*Distribution of Campylobacter infection cases*

The number of *Campylobacter* infections notified in the national platform SINAVE has been rising over time, contrary to what has been observed at the European level, where notification rates remained stable in the five-year period preceding the COVID-19 pandemic [1]. Despite the overt growth of notified confirmed cases (from 596 to 973 in the period 2017–2021), the notification rate remained far below expectations peaking at 9.4 cases per 100,000 population in 2021, less than a quarter of the European Union (EU)/European Economic Area (EEA) overall notification rate of 44.5 cases per 100,000 population that year [21]. Thus, neither the increase in notifications can be assumed as a direct increase in *Campylobacter* infections, as there have been improvements in laboratory testing and reporting over time, nor the number of annual cases can be accurately estimated since there is evidence of a considerable rate of under-notification. As expected, the dynamics of the number of isolates sent to the NRL followed the same trend as the number of notifications; however, contrary to expectations, until 2021, the number of isolates received from the sentinel surveillance was almost as high as the cases notified to a national level, reinforcing the extent of under-notification of campylobacteriosis in Portugal. Due to the very high load of isolates received, a pre-selection step in the laboratory protocol was introduced, in 2019, resulting in a decrease in cases processed by the NRL, which was further noticed by the impact of the COVID-19 pandemic in 2020.

*Seasonal variation of Campylobacter infection*

The seasonal distribution of *Campylobacter* infection cases exhibited a unique pattern, with a lack of clear summer peaks usually observed in the EU [4], despite the highest cumulative number of cases occurring in August. Instead, a more random distribution throughout the year was noted, with slight decreases in the winter months. Reinforcing this random distribution was the occurrence of infection peaks in months with usually lower number of cases, such as October and February. Several studies have been carried out aiming to understand the underlying determinants of the marked campylobacteriosis seasonality [22]; however, epidemiological explanations remain uncertain [23]. This highlights the potential of WGS to provide deeper insights into strain-specific dynamics and transmission patterns.

*Epidemiological information*

The study emphasizes a significant predominance of campylobacteriosis cases among the pediatric population (77.7%, 3979/5120), deviating from the European trend where adults constitute the majority of cases [1]. Considering the data on the notification rates of campylobacteriosis, it is possible to verify a very marked disparity between the values reported in Portugal and the overall values observed in the EU. However, looking at these values by age group, it is shown that this disparity is particularly focused on the adult population, with the pediatric population approaching the average over the years. In fact, in 2021, the notification rate in children under five years of age in Portugal surpassed the overall notification rate in the EU/EEA [21]. Thus, it is plausible to assume that, despite the extensive underreporting in Portugal, this is substantially more relevant in the adult population. This disparity could be attributed to differences in clinical awareness, reporting practices, and severity of symptoms between adults and children. Indeed, an association between bloody diarrhea and the pediatric population under four years old was observed, suggesting potential differences in disease manifestation across age groups.

In this analysis, data relating to age groups showed a subdivision of children under one year of age. Although generally not used, thus not allowing for a comparison with data obtained in the EU, this division highlights a relevant number of cases at an age where direct transmission via food is not expected. Thus, the observed high values may be indicators of the impact of poor food preparation measures, resulting in cross-contamination, and of exposure of very young children to untreated water, such as private drinking water wells in more rural areas [24,25,26,27,28].

Regarding gender distribution, 60.2% (3096/5144) of the patients were male, denoting a statistically significant association, in accordance with what has been described throughout the EU [1].

Additionally, molecular typing data of *Campylobacter* spp. isolated from 2016 to 2021 revealed a higher genetic diversity of *C. jejuni* circulating in Portugal in comparison with *C. coli*, as shown by the differences in the number of observed MLST clonal complexes. The high prevalence of ST-828 clonal complex strains in this study is in agreement with previous observations throughout the world [29,30]. Nevertheless, the number of STs was high for both species (77 STs for *C. jejuni* and 47 STs for *C. coli*), which was distributed over the studied years (2016 to 2021). Likewise, the high genetic diversity hampered establishing an association between ST and other demographic data, such as age group.

*Antimicrobial resistance*

Antibiotic resistance in *Campylobacter* spp. is an increasingly serious threat due to its implications for public health and effective treatment, requiring coordinated actions to minimize the emergence and spread of antimicrobial-resistant strains. Ciprofloxacin and erythromycin had been considered the antibiotics of choice for treating *Campylobacter* infections until very recently when an increasing trend in ciprofloxacin resistance has been observed [2,31]. In contrast, resistance to erythromycin and aminoglycosides like gentamicin remains very low [1]. Thus, monitoring resistance levels in circulating isolates becomes one of the most relevant parameters for the surveillance of campylobacteriosis. Patterns of AMR in *Campylobacter* isolates from Portugal revealed critical levels of resistance to priority antibiotics, particularly in *C. coli*. This is in line with what has already been described in association with the cost fitness that mutations associated with resistance, especially toward macrolides, have in *C. jejuni* and not in *C. coli* [32]. Overall, *C. coli* presented an extremely high level of resistance to both ciprofloxacin and tetracycline, and a high level to erythromycin, while *C. jejuni* presented an extremely high level of resistance to ciprofloxacin and a very high level to tetracycline only. Although rare in *C. jejuni* (2.7%), MDR was frequently found in *C. coli* isolates (50.5%) and is of concern for both species. Indeed, the occurrence of combined resistance to fluoroquinolones and macrolides in *Campylobacter* spp. is considered of high public health relevance [33]. These patterns of AMR, observed both in *C. jejuni* and in *C. coli* human isolates, are of the highest among EU member states, according to the latest EU summary reports on AMR in zoonotic and indicator bacteria, and are in line with data from animal and food isolates [33].

Temporal analysis of resistance to priority antibiotics demonstrated stability, with slight fluctuations, over the years, such as a momentary decrease in erythromycin resistance in 2020, potentially linked to COVID-19-related restrictions [33]. However, given the fact that Portugal has come to be the European country with the highest levels of resistance to this antibiotic in both people and animals, it reduces the likelihood that the generally observed levels of resistance derive from travel-related cases. With the misuse of antibiotics in veterinary medicine pointed out as the main cause of the historical increase in the level of resistance, the banning and control of their use has not been accompanied by a decrease in the observed levels of AMR, which in part could be explained by the stability of the resistance phenotype that would remain even without antibiotic pressure, if not presenting biological costs [34]. Our data showed that WGS could predict antimicrobial resistance with high precision. Therefore, AMR surveillance through WGS can be a valuable addition to, or a replacement of, the phenotypic surveillance, providing insights into the genetic basis of resistance mechanisms, as well monitoring the emergence and spread of MDR clones [35,36]. In the present study, for *C. coli*, a distribution of the genetic resistance markers was observed among the different STs circulating over the studied time period, challenging the notion of a clonal origin for MDR isolates, and explaining the high rates of resistance observed for this species, regarding the priority antibiotics ciprofloxacin, erythromycin, and tetracycline (50.5% of MDR). This same scenario was observed for *C. jejuni* regarding the antibiotics ciprofloxacin and tetracycline, where the dispersion of resistance determinants matched the high rate of resistant strains. As for erythromycin, only a small fraction of the typed isolates harbored the associated point mutations in the *23S rRNA* gene, matching with an overall low resistance rate to this antibiotic observed in *C. jejuni* (3.3%). Half of these belong to ST10662, which might indicate a clonal origin for the MDR strains, also corroborating a much lower resistance rate, when compared to *C. coli*.

The monitoring of optional listed antibiotics is particularly relevant in countries with high rates of AMR, as is the case in Portugal, since they may represent the only treatment option in cases of infection with MDR strains [37]. While most of the strains displayed resistance to ampicillin, only a very low to low proportion of strains displayed resistance to ertapenem and decreased susceptibility to amoxicillin–clavulanic acid. Resistance to carbapenems, despite their observed levels being low so far, represents a public health concern, as it is a recommended treatment option in more severe cases of systemic infections [38,39]. Thus, and taking into account the high levels of resistance recently observed in *C. coli* isolated from animal sources, it becomes relevant to define the cut-off for ertapenem, as well as the harmonization of its surveillance both in *C. coli* and in *C. jejuni* [33]. Again, WGS is of most value to unravel the mechanisms behind resistance to β-lactams and carbapenems and to monitor the evolution of resistant clones [40].

Associations between demographic features and antimicrobial resistance were observed, particularly a reduced likelihood of resistance in the 15–44 years and 45+ age groups. Geographic distribution also showed relevance, with certain resistance patterns more prevalent in specific regions. These findings could reflect differences in exposure routes, emphasizing the importance of targeted interventions.

## 5. Conclusions

In conclusion, this study provides valuable insights into campylobacteriosis surveillance in Portugal, identifying trends, disparities, and areas of concern. While limitations in reporting and laboratory processing are evident, the data underscore the importance of sustained surveillance but also calls for a page turn, with the routine implementation of WGS-based surveillance for a more comprehensive understanding of disease dynamics and AMR evolution. The findings emphasize the urgent need to implement a One Health approach to effectively combat campylobacteriosis and its associated challenges in Portugal.

## Figures and Tables

**Table 1:** Total number of cases of *Campylobacter* infections received in the National Reference Laboratory, and total number of non-duplicated isolates processed, including with antimicrobial susceptibility testing and whole-genome sequencing performed.

	Year													Overall
	2009	2010	2011	2012	2013	2014	2015	2016	2017	2018	2019	2020	2021	
Total cases received in the NRL	129	248	347	328	426	437	461	537	685	658	916	676	820	6668
Isolates processed by the NRL	129	248	347	328	426	437	461	537	685	658	364	260	325	5205
Isolates with antibiotic susceptibility data	0	0	0	0	119	135	182	191	289	336	349	248	325	2174
Isolates with typing data *	0	0	0	0	0	0	0	20/17	23/30	27/42	21/32	22/53	23/70	380

* Typing data numbers refer to *C. coli*/*C. jejuni* isolates for which whole-genome sequencing was performed and in silico data for MLST profile and resistance determinants were retrieved; NRL—National Reference Laboratory.

**Table 2:** Distribution of *Campylobacter* species by year.

	Year													Overall
	2009	2010	2011	2012	2013	2014	2015	2016	2017	2018	2019	2020	2021	
*C. jejuni* *n* (%)	112 (86.8)	208 (83.9)	300 (86.5)	294 (89.6)	393 (92.3)	388 (88.8)	394 (85.5)	474 (88.3)	626 (91.4)	591 (89.8)	311 (85.4)	221 (85.0)	278 (85.5)	4590 (88.2)
*C. coli* *n* (%)	17 (13.2)	40 (16.1)	47 (13.5)	34 (10.4)	33 (7.7)	49 (11.2)	67 (14.5)	63 (11.7)	59 (8.6)	67 (10.2)	53 (14.6)	39 (15.0)	47 (14.5)	615 (11.8)
Total	129	248	347	328	426	437	461	537	685	658	364	260	325	5205

**Table 3:** Distribution of the main symptoms associated with *Campylobacter* infections.

Reported Symptoms	With Diarrhea (Including Bloody Diarrhea) *n* = 3405 *n* (% within Group)	Without Diarrhea *n* = 268 *n* (% within Group)
Abdominal pain	299 (8.8)	65 (24.3)
Fever	316 (9.3)	58 (21.6)
Vomiting	102 (3.0)	12 (4.5)
Abdominal pain + fever	61 (1.8)	14 (5.2)
Abdominal pain + vomiting	27 (0.8)	7 (2.6)
Abdominal pain + fever + vomiting	12 (0.4)	2 (0.7)
Fever + vomiting	68 (2.0)	2 (0.7)
No other reported symptoms	2520 (74.0)	--

**Table 4:** Distribution of the main symptoms among pediatric and adult population.

Main Symptom	Pediatric Population (*n* = 2982) *n* (% within Group)	Adult Population (*n* = 677) *n* (% within Group)	Total (*n* = 3659) *n* (% of Total)	OR (95%CI)	*p*
Bloody Diarrhea	1353 (45.4)	101 (14.9)	1454 (39.7)	4.7367 (3.788–5.922)	<0.001
Non-bloody diarrhea	1492 (48.4)	449 (66.3)	1941 (53.0)	0.5085 (0.427–0.606)	<0.001
Abdominal pain	345 (11.6)	140 (20.7)	485 (13.3)	0.5018 (0.404–0.624)	<0.001
Fever	404 (13.5)	128 (18.9)	592 (16.2)	0.6721 (0.540–0.837)	<0.001
Vomiting	180 (7.3)	52 (7.7)	232 (6.3)	0.7721 (0.560–1.064)	0.114

**Table 5:** Number of *Campylobacter* infections in the studied population according to age, gender, and geographic region.

	No. Positive Samples (% within Group) (% of Total)		Total
	*Campylobacter jejuni*	*Campylobacter coli*	
Gender (*n* = 5144)			
Female	1791 (39.5 ^a^) (34.8)	257 (42.3 ^a^) (5.0)	2048 (39.8)
Male	2745 (60.5 ^a^) (53.4)	351 (57.7 ^a^) (6.8)	3096 (60.2)
Total	4536 (88.2)	608 (11.8)	5144 (100)
Age group (*n* = 5120)			
<1	831 (18.4 ^a^) (16.2)	103 (17.0 ^a^) (2.0)	934 (18.2)
1–4	1807 (40.0 ^a^) (35.3)	194 (32.1 ^b^) (3.8)	2001 (39.1)
5–9	589 (13.0 ^a^) (11.5)	68 (11.2 ^a^) (1.3)	657 (12.8)
10–14	348 (7.7 ^a^) (6.8)	36 (6.0 ^b^) (0.7)	384 (7.5)
15–44	482 (10.7 ^a^) (9.4)	95 (15.7 ^b^) (1.9)	577 (11.3)
45–64	212 (4.7 ^a^) (4.1)	52 (8.6 ^b^) (1.0)	264 (5.2)
+ 65	246(5.4 ^a^) (4.8)	57 (9.4 ^b^) (1.1)	303 (5.9)
Total	4515 (88.2 ^a^)	605 (11.8 ^b^)	5120 (100)
Region (*n* = 5203)			
North	2317 (50.5 ^a^) (45.0)	271(44.1 ^b^) (5.2)	2588 (44.7)
Center	711 (15.5 ^a^) (13.9)	78 (12.7 ^a^) (1.5)	789 (15.2)
Metropolitan Lisbon Area	1560 (34.0 ^a^) (29.5)	241 (43.3 ^b^) (5.1)	1826 (35.1)
Total	4588 (88.2)	615 (11.8)	5203 (100)

Prevalence (in %) in columns with different letters (a and b) are significantly different (*p* < 0.05).

**Table 6:** Resistance profile to seven antimicrobial agents of *Campylobacter jejuni* and *Campylobacter coli* strains.

Antimicrobial Category	Antimicrobial Agent	Mean Inhibition Diameter Zone (in mm) (SD)			*p*	% Resistance (*n*/*n* Total)			*p*
		Total	*C. jejuni*	*C. coli*		Total (*n* = 2174)	*C. jejuni* (*n* = 1807)	*C. coli* (*n* = 367)	
Fluoroquinolones	Ciprofloxacin (CIP)	9.35 (7.07)	10.54 (8.70)	8.66 (579)	0.002	94.2 (2048)	93.7 (1694)	96.5 (354)	0.043
Macrolides	Erythromycin (ERY)	26.31 (8.02)	28.12 (5.44)	15.85 (11.06)	<0.001	11.8 (252)	3.3 (60)	52.3 (192)	<0.001
Tetracyclines	Tetracycline (TCY)	14.36 (11.53)	16.66 (12.78)	10.02 (7.97)	<0.001	81.6 (1773)	79.2 (1431)	93.2 (342)	<0.001
Aminoglycosides	Gentamicin (GEN)	28.40 (3.95)	28.47 (3.78)	26.69 (4.21)	<0.001	0.5 (11)	0.1 (2)	2.5 (9)	<0.001
Penicillins + β-lactamase inhibitors	Amoxicillin–clavulanic acid (AMC) *	28.66 (4.71)	28.76 (4.34)	23.95 (5.79)	<0.001	0.8 (12/1547)	0.2 (3/1314)	3.9 (9/233)	<0.001
Carbapenems	Ertapenem (ETP) **	0.125 (0.019)	0.19 (0.034)	0.5 (0.075)	<0.001	1.6 (24/1543)	0.7 (9/1311)	6.5 (15/232)	<0.001
Penicillins	Ampicillin (AMP)	10.42 (7.22)	11.56 (7.64)	10.69 (7.47)	0.348	76.3 (944/1238)	75.5 (794/1051)	80.2 (150/187)	0.192

* For AMC, the % of resistance corresponds to % of decreased susceptibility or intermediate category, according to [16]. ** For ERT, the values correspond to the minimal inhibitory concentration, in mg/L. *p* values refer to differences between *Campylobacter jejuni* and *Campylobacter coli*.

**Table 7:** Multivariate logistic regression analysis of the relationship between *Campylobacter jejuni* and *Campylobacter coli* antibiotic resistance and sociodemographic features.

	Amoxicillin			Ampicillin			Ciprofloxacin			Erythromycin			Ertapenem			Gentamicin			Tetracycline		
	R	S	*p* Value OR (95%CI)	R	S	*p* Value OR (95%CI)	R	S	*p* Value OR (95%CI)	R	S	*p* Value OR (95%CI)	R	S	*p* Value OR (95%CI)	R	S	*p* Value OR (95%CI)	R	S	*p* Value OR (95%CI)
Gender Female Male	6 6	614 916	0.697 Ref 0.793 (0.247–2.547)	372 568	127 167	0.279 Ref 1.160 (0.887–1.515)	810 1225	48 78	0.734 Ref 0.937 (0.645–1.363)	112 138	1185 746	0.959 Ref 0.991 (0.714–1.377)	11 13	605 909	0.662 Ref 0.831 (0.362–1.907)	6 5	852 1298	0.470 Ref 0.640 (0.190–2.150)	708 1055	150 248	0.509 Ref 0.926 (0.736–1.164)
Age <15 15–44 45+	8 2 2	960 302 267	0.825 Ref 0.658 (0.134–3.234) 0.700 (0.143–3.425)	589 184 168	160 67 67	0.022 Ref 0.714 (0.510–1.000) 0.660 (0.472–0.923)	1344 355 337	63 30 33	0.001 Ref. 0.531 (0.335–0.841) 0.456 (0.293–0.709)	148 44 57	1259 341 313	0.183 Ref. 1.002 (0.646–1.553) 1.458 (0.965–2.202)	16 5 3	948 299 267	0.564 Ref. 0822 (0.291–2.322) 0.506 (0.143–1.790)	7 2 2	1400 383 368	0.949 Ref. 0.850 (0.171–4.230) 0.787 (0.158–3.916)	1178 308 278	229 77 92	<0.001 Ref. 0.778 (0.579–1.046) 0.571 (0.431–0.756)
Species *C. jejuni* *C. coli*	3 9	1311 224	<0.001 Ref. 17.987 (4.765–67.90)	794 150	257 37	0.123 Ref. 1.360 (0.920–2.010)	1694 354	113 13	0.041 Ref. 1.850 (1.026–3.336)	60 192	1747 175	<0.001 Ref. 31.088 (22.27–43.40)	9 15	1302 217	<0.001 Ref. 9.775 (4.193–22.79)	2 9	1805 358	<0.001 Ref. 20.989 (4.48–98.33)	1431 342	376 25	<0.001 Ref. 3.705 (2.423–5.666)
Region North Center Metropolitan Lisbon Area	5 3 4	690 176 668	0.390 Ref. 1.994 (0.447–8.901) 0.680 (0.177–2.614)	403 112 428	136 32 126	0.421 Ref. 1.206 (0.773–1.882) 1.195 (0.901–1.585)	940 227 880	59 20 47	0.166 Ref. 0.767 (0.449–1.310) 1.276 (0.854–1.908)	98 35 119	901 212 808	0.698 Ref. 1.205 (0.714–2.034) 0.966 (0.680–1.373)	6 2 16	686 177 655	0.135 Ref. 1.081 (0.21–5.556) 2.480 (0.945–6.513)	3 1 7	996 246 920	0.574 Ref. 1.151 (0.116–11.47) 2.023 (0.507–8.066)	828 203 741	171 44 186	0.215 Ref. 1.077 (0.741–1.564) 1.236 (0.974–1.568)

## Data Availability

The other data will be made available on request.

## Supplementary Materials

The following supporting information can be downloaded at: https://www.mdpi.com/article/10.3390/pathogens13020147/s1, Seven supplementary tables are provided in ‘Supplementary Material’ to support additional findings produced from the present extensive analysis and the relevant contents of these tables are indicated in ‘Results’. Table S1: Distribution (odds ratio and confidence intervals) of a main symptom associated with *Campylobacter infection* by age group. Table S2: Risk ratio of cases of *Campylobacter* infection (male/female) by age group. Table S3: Distribution of *Campylobacter* species for pediatric (<15 years old) and adult population by year. Table S4: Time (in days) elapsed from onset of symptoms to the date of sample collection according to gender and age group. Table S5: Evolution of resistance (%) to different antimicrobial agents for *Campylobacter jejuni* and *Campylobacter coli*. Table S6: Univariate logistic regression analysis of the relationship between *Campylobacter jejuni* and *Campylobacter coli* resistance and sociodemographic features. Table S7: Genomic data (MLST type and antimicrobial resistance genetic marker) of the *Campylobacter* spp. isolates enrolled in the present study.
