# Supplementary material for: Epidemiological Data and Antimicrobial Resistance of Campylobacter spp. in Portugal from 13 Years of Surveillance
Source: Pathogens. 2024 Feb 6;13(2):147. doi: 10.3390/pathogens13020147 (PMC10893263; doi:10.3390/pathogens13020147)
Supplement: Supplementary file 1 [file pathogens-13-00147-s001.zip › pathogens-2797597-supplementary.pdf]

Table S1. Distribution (odds ratio and confidence intervals) of a main symptom associated with *Campylobacter* infection by age group.

	**Bloody diarrhoea**			**Non-bloody Diarrhoea**			**Abdominal pain**			**Fever**			**Vomiting**		
	Yes	No	p-value OR (95%CI)	Yes	No	p-value OR (95%CI)	Yes	No	p-value OR (95%CI)	Yes	No	p-value OR (95%CI)	Yes	No	p-value OR (95%CI)
**Age**			<0.001			<0.001			<0.001			<0.001			<0.001
<1	368	346	Ref.	323	390	Ref.	47	666	Ref.	82	633	Ref.	38	677	Ref.
1-4	699	812	0.809 (0.677-0.967)	757	754	1.212 (1.014-1.449)	151	1360	1.573 (1.120-2.211)	206	1310	1.214 (0.924-1.595)	71	1445	0.875 (0.584-1.312)
5-9	198	295	0.631 (0.500-0.796)	269	224	1.450 (1.151-1.826)	85	410	2.938 (2.015-4.283)	67	429	1.206 (0.854-1.703)	45	451	1.778 (1.136-2.782)
10-14	88	176	0.470 (0.350-0.631)	143	120	1.44 (1.083-1.911)	63	200	4.464 (2.964-6.722)	49	215	1.759 (1.196-2.709)	26	238	1.946 (1.157-3.274)
15-44	78	269	0.273 (0.204-0.365)	200	147	1.643 (1.268-2.128)	84	264	4.509 (3.070-6.622)	69	279	1.909 (1.346-2.274)	23	325	1.261 (0.739-2.152)
45-64	19	143	0.125 (0.076-0.206)	112	50	2.705 (1.878-3.894)	38	124	4.392 (2.718-6.938)	35	128	2.111 (1.361-3.274)	13	150	1.544 (0.803-2.970)
65+	4	164	0.023 (0.008-1.063)	137	31	5.386 (3.517-8.095)	18	150	1.7 (0.960-3.011)	24	144	1.287 (0.789-2.099)	16	152	1.875 (1.019-3.452)

Table S2. Risk ratio of cases of *Campylobacter* infection (male/female) by age group.

	Age group						
	**<1**	**1-4**	**5-9**	**10-14**	**15-44**	**45-64**	**65+**
**RR**	0.931	1.065	1.026	1.144	0.799	0.998	0.935
**(95% CI)**	(0.828-1.047)	(0.992-1.187)	(0.886-1.148)	(0.937-1.397)	(0.684-0.933)	(0.782-1.274)	(0.749-1.167)
**p-value**	0.2333	0.0802	0.7351	0.1876	0.0045	0.988	0.5506

Table S3. Distribution of *Campylobacter* species for paediatric (<15 years old) and adult population by year.

		No. (% within group)													
		**2009**	**2010**	**2011**	**2012**	**2013**	**2014**	**2015**	**2016**	**2017**	**2018**	**2019**	**2020**	**2021**	**Overall**
**Paediatric population**	***C. jejuni***	75 (87.2)	185 (86.4)	271 (87.4)	258 (89.9)	350 (91.9)	293 (90.4)	313 (87.7)	383 (90.3)	502 (93.1)	456 (92.3)	200 (88.9)	137 (85.6)	152 (86.9)	3576 (89.9)
	C. coli	11 (12.8)	29 (13.6)	39 (12.6)	29 (10.1)	31 (8.1)	31 (9.6)	44 (12.3)	41 (9.7)	37 (6.9)	38 (7.7)	25 (11.1)	23 (14.4)	23 (13.1)	401 (10.1)
	**Total**	86	214	310	287	381	324	357	424	539	494	225	160	175	3976
**Adult population**	***C. jejuni***	15 (71.4)	19 (73.1)	28 (77.8)	35 (87.5)	37 (94.9)	73 (83.0)	76 (76.8)	89 (80.9)	120 (85.1)	127 (81.9)	111 (79.9)	84 (84.0)	126 (84.0)	940 (82.2)
	C. coli	6 (28.6)	7 (26.9)	8 (22.2)	5 (12.5)	2 (5.1)	15 (17.0)	23 (23.2)	21 (19.1)	21 (14.9)	28 (18.1)	28 (20.1)	16 (16.0)	24 (16.0)	24 (16.0)
	**Total**	21	26	36	40	39	88	99	110	141	155	139	100	150	1144

Table S4. Time (in days) elapsed from onset of symptoms to the date of sample collection according to gender and age group.

	Time (days)	
	**Mean (SD)**	**Median (IQR)**
**Gender**		
Male	4.52 (6.818)	3 (2-5)
Female	5.49 (9.049)	4 (2-6)
P		0.014
**Age group (years)**		
<1	4.71 (8.099)	3 (1-5)
1-4	5.08 (9.054)	4 (2-5)
5-9	4.50 (5.108)	3 (2-5)
10-14	4.47 (5.574)	3.5 (2-5)
15-44	5.40 (7.128)	3 (2-5)
45-64	5.63 (6.113)	3 (3-7.5)
65+	5.63 (6.927)	3 (2-6.25)
P		0.896

Table S5. Evolution of resistance (%) to different antimicrobial agents for *Campylobacter jejuni* and *Campylobacter coli*.

	2013		2014		2015		2016		2017		2018		2019		2020		2021	
**Antibiotic**	***C. jejuni*** **(n=97)**	***C. coli*** **(n=22)**	***C. jejuni*** **(n=103)**	***C. coli*** **(n=32)**	***C. jejuni*** **(n=140)**	***C. coli*** **(n=42)**	***C. jejuni*** **(n=153)**	***C. coli*** **(n=38)**	***C. jejuni*** **(n=246)**	***C. coli*** **(n=43)**	***C. jejuni*** **(n=280)**	***C. coli*** **(n=56)**	***C. jejuni*** **(n=298)**	***C. coli*** **(n=51)**	***C. jejuni*** **(n=212)**	***C. coli*** **(n=36)**	***C. jejuni*** **(n=256)**	***C. coli*** **(n=47)**
**Ciprofloxacin (CIP)**	89.7	90.9	98.1	96.9	97.9	100	94.1	100	96.3	97.7	94.6	94.6	93.0	96.1	89.6	88.9	92.1	100
**Erythromycin (ERY)**	4.1	45.5	4.9	43.8	3.6	50.0	3.9	44.7	2.8	53.5	5.4	57.1	2.3	72.5	0.9	36.1	3.2	53.2
**Tetracycline (TCY)**	70.1	86.4	77.7	87.5	82.9	92.9	79.7	92.1	84.1	88.4	82.5	96.4	77.5	98.0	73.6	88.9	79.1	100
**Gentamicin (GEN**	0	9.1	0	0	0	2.4	0	0	0	2.3	0.4	3.6	0.3	3.9	0	0	0	2.1
**Amoxicillin-clavulanic acid (AMC)**	-	-	-	-	-	-	-	-	0	2.3	1.1	14.3	0	0	0	0	0	0
**Ertapenem (ETP)**	-	-	-	-	-	-	-	-	0.4	9.3	1.4 (4/277)	3.6 (2/55)	0.7	5.9	0.5	5.6	0.4	8.5
**Ampicillin (AMP)**	-	-	-	-	-	-	-	-	-	-	79.6 (203/264)	67.9 (36/53)	77.2	88.2	66.8	80.6	79.1	85.1

Table S6. Univariate logistic regression analysis of the relationship between *Campylobacter jejuni* and *Campylobacter coli* resistance and sociodemographic features.

	**Amoxicillin**			**Ampicillin**			**Ciprofloxacin**			**Erythromycin**			**Ertapenem**			**Gentamicin**			**Tetracycline**		
	R	S	P value OR (95%CI)	R	S	P value OR (95%CI)	R	S	P value OR (95%CI)	R	S	P value OR (95%CI)	R	S	P value OR (95%CI)	R	S	P value OR (95%CI)	R	S	P value OR (95%CI)
**Gender**			0.490			0.269			0.704			0.08			0.561			0.320			0.363
Female	6	614	Ref	372	127	Ref	810	48	Ref	112	1185	Ref	11	605	Ref	6	852	Ref	708	150	Ref
Male	6	916	0.670	568	167	1.161	1225	78	0.931	138	746	0.789	13	909	0.787	5	1298	0.547	1055	248	0.901
			(0.215-2.088)			(0.661-1.121)			(0.643-1.348)			(0.605-1.029)			(0.350-1.767)			(0.166-1.798)			(0.720-1.128)
**Age**			0.956			**0.040**			**0.001**			**0.034**			0.809			0.994			**<0.001**
<15	8	960	Ref	589	160	Ref	1344	63	Ref.	148	1259	Ref.	16	948	Ref.	7	1400	Ref.	1178	229	Ref.
15-44	2	302	0.795	184	67	0.746	355	30	0.555	44	341	1.098	5	299	0.991	2	383	1.044	308	77	0.778
			(0.168-3.763)			(0.536-1.037)			(0.354-0.870)			(0.768-1.569)			(0.360-2.727)			(0.216-5.048)			(0.584-1.036)
45+	2	267	0.899			0.681			0.479	57	313	1.549	3	267	0-666	2	368	1.087	278	92	0.587
			(0.19-4.258)	168	67	(0.488-0.950)	337	33	(0.309-0.342)			(1.114-2.154)			(0.193-2.302)			(0.225-5.254)			(0.446-0.773)
**Species**			**<0.001**			0.168			**0.046**			**<0.001**			**<0.001**			**<0.001**			**<0.001**
C. jejuni	3	1311	Ref.	794	257	Ref.	1694	11	Ref.	60	1747	Ref.	9	1302	Ref.	2	1805	Ref.	1431	376	Ref.
C. coli	9	224	17.558	150	37	1.312	354	3	1.816	192	175	31.945	15	217	10.0	9	358	22.689	342	25	3.594
			(4.717-65.354)			(0.892-1.931)		13	(1.012-3.262)			(22.99-44.39)			(4.323-23.14)			(4.88-105.45)			(2.358-5.48)
**Region**			0.360			0.563			0.195			**0.048**			0.083			0.386			0.241
North	5	690	Ref.	403	136	Ref.	940	59	Ref.	98	901	Ref.	6	686	Ref.	3	996	Ref.	828	171	Ref.
Center	3	176	2.352			1.181			0.712			1.518			1.292			1.350			0.953
			(0.557-9.937)	112	32	(0.762-1.831)	227	20	(0.420-1.207)	35	212	(1.003-2.296)	2	177	(0.259-6.455)	1	246	(0.14-13.03)	203	44	(0.661-1.373)
Lisbon	4	668	0.826	428	126	1.146	880	47	1.175	119	808	1.354	16	655	2.793	7	920	2.526	741	186	0.823
and TV			(0.221-3.091)			(0.868-1.514)			(0.792-1.743)			(1.020-1.798)			(1.086-7.18)			(0.651-9.798)			(0.654-1.036)

Table S7. Genomic data (MLST Type and antimicrobial resistance genetic marker) of the Campylobacter spp. isolates enrolled in the present study. NA - Not applicable; NF - Not found; MLST – Multi-locus Sequence Type; ENA – European Nucleotide Archive; AMRg - Antimicrobial resistance genetic marker.

Isolate ID	Species	Isolation year	MLST	ST clonal_complex	Ciprofloxacin AMRg	Tetracycline AMRg	Streptomycin AMRg	Erythromycin AMRg	ENA Accession Number
C001	Campylobacter coli	2016	8104	ST-828 complex	T86I	tet(O)	NF	A2075G	ERR10372370
C002	Campylobacter coli	2016	828	ST-828 complex	T86I	tet(O)	aadE-Cc	A2075G	ERR10372369
C003	Campylobacter coli	2016	7345	ST-828 complex	T86I	tet(O)	NF	A2075G	ERR10372306
C004	Campylobacter coli	2016	9449	ST-1150 complex	T86I	tet(O)	NF	A2075G	ERR10372422
C005	Campylobacter coli	2016	11397	ST-828 complex	T86I	tet(O)	aadE-Cc	A2075G	ERR10372480
C006	Campylobacter coli	2016	1585	ST-828 complex	T86I	NF	aadE-Cc	A2075G	ERR10372417
C007	Campylobacter coli	2016	2177	ST-828 complex	T86I	tet(O)	NF	A2075G	ERR10372410
C008	Campylobacter coli	2016	3016	ST-828 complex	T86I+D90N	tet(O)	NF	A2075G	ERR10372425
C009	Campylobacter coli	2016	832	ST-828 complex	T86I	tet(O)	NF	A2075G	ERR10372335
C010	Campylobacter coli	2016	3016	ST-828 complex	T86I	tet(O)	NF	A2075G	ERR10372340
C011	Campylobacter coli	2016	899	ST-828 complex	T86I	tet(O)	NF	A2075G	ERR10372359
C012	Campylobacter coli	2016	11395	NA	T86I	tet(O)	NF	A2075G	ERR10372445
C013	Campylobacter coli	2016	855	ST-828 complex	T86I	tet(O)	NF	A2075G	ERR10372397
C014	Campylobacter coli	2016	6820	ST-1150 complex	T86I	tet(W)	NF	A2075G	ERR10372398
C015	Campylobacter coli	2016	902	ST-828 complex	T86I	tet(O)	aadE-Cc	A2075G	ERR10372401
C016	Campylobacter coli	2016	8104	ST-828 complex	T86I	tet(O)	NF	A2075G	ERR10372413
C017	Campylobacter coli	2016	10838	ST-1150 complex	T86I	tet(W)	NF	A2075G	ERR10372429
C018	Campylobacter coli	2016	3016	ST-828 complex	T86I	tet(O)	NF	A2075G	ERR10372420
C019	Campylobacter coli	2016	828	ST-828 complex	T86I	tet(O)	aadE-Cc	A2075G	ERR10372433
C020	Campylobacter coli	2016	1413	ST-828 complex	T86I	tet(O)	aadE-Cc	A2075G	ERR10372408
C021	Campylobacter coli	2017	10836	ST-828 complex	T86I	tet(O)	NF	A2075G	ERR10372475
C022	Campylobacter coli	2017	11396	ST-828 complex	T86I	tet(O)	NF	A2074N	ERR10372395
C023	Campylobacter coli	2017	825	ST-828 complex	T86I	tet(O)	NF	A2075G	ERR10372437
C024	Campylobacter coli	2017	3016	ST-828 complex	T86I	tet(O)	NF	A2075G	ERR10372461
C025	Campylobacter coli	2017	832	ST-828 complex	T86I	tet(O)	NF	A2075G	ERR10372447

C026	Campylobacter coli	2017	832	ST-828 complex	T86I	tet(O)	NF	A2075G	ERR10372347
C027	Campylobacter coli	2017	3016	ST-828 complex	T86I	tet(O)	NF	A2075G	ERR10372470
C028	Campylobacter coli	2017	3017	ST-828 complex	T86I	tet(O)	NF	A2075G	ERR10372414
C029	Campylobacter coli	2017	6233	ST-828 complex	T86I	tet(O)	NF	A2074N	ERR10372464
C030	Campylobacter coli	2017	3017	ST-828 complex	T86I	tet(O)	NF	A2075G	ERR10372348
C031	Campylobacter coli	2017	3017	ST-828 complex	T86I	tet(O)	NF	A2075G	ERR10372333
C032	Campylobacter coli	2017	832	ST-828 complex	T86I	tet(O)	NF	A2075G	ERR10372346
C033	Campylobacter coli	2017	860	ST-828 complex	T86I	tet(O)	NF	A2075G	ERR10372321
C034	Campylobacter coli	2017	832	ST-828 complex	T86I	tet(O)	NF	A2075G	ERR10372385
C035	Campylobacter coli	2017	860	ST-828 complex	T86I	tet(O)	NF	A2075G	ERR10372378
C036	Campylobacter coli	2017	2177	ST-828 complex	T86I	tet(O)	NF	A2075G	ERR10372383
C037	Campylobacter coli	2017	832	ST-828 complex	T86I	tet(O)	NF	A2075G	ERR10372402
C038	Campylobacter coli	2017	3017	ST-828 complex	T86I	tet(O)	NF	A2075G	ERR10372419
C039	Campylobacter coli	2017	3017	ST-828 complex	T86I	tet(O)	NF	A2075G	ERR10372444
C040	Campylobacter coli	2017	860	ST-828 complex	T86I	tet(O)	NF	A2075G	ERR10372305
C041	Campylobacter coli	2017	860	ST-828 complex	T86I	tet(O)	NF	A2075G	ERR10372495
C042	Campylobacter coli	2017	860	ST-828 complex	T86I	tet(O)	NF	A2075G	ERR10372354
C060	Campylobacter coli	2018	3016	ST-828 complex	T86I	tet(O)	NF	A2075G	ERR10372466
C061	Campylobacter coli	2018	3016	ST-828 complex	T86I	tet(O)	NF	A2075G	ERR10372357
C062	Campylobacter coli	2018	9630	NA	NF	NF	NF	NF	ERR10372460
C063	Campylobacter coli	2018	1239	NA	T86I	tet(O)	NF	A2075G	ERR10372481
C064	Campylobacter coli	2018	828	ST-828 complex	T86I	tet(O)	aadE-Cc	A2075G	ERR10372352
C065	Campylobacter coli	2018	832	ST-828 complex	T86I	tet(O)	NF	A2075G	ERR10372485
C066	Campylobacter coli	2018	1595	ST-828 complex	T86I	tet(O)	ant(6)-Ia	NF	ERR10372327
C067	Campylobacter coli	2018	10844	ST-828 complex	T86I	tet(O)	NF	A2075G	ERR10372336
C068	Campylobacter coli	2018	832	ST-828 complex	T86I	tet(O)	NF	A2075G	ERR10372424
C069	Campylobacter coli	2018	10844	ST-828 complex	T86I	tet(O)	NF	A2075G	ERR10372318
C070	Campylobacter coli	2018	832	ST-828 complex	T86I+D90N	tet(O)	NF	A2075G	ERR10372454
C071	Campylobacter coli	2018	8104	ST-828 complex	T86I	tet(O)	NF	A2075G	ERR10372324
C072	Campylobacter coli	2018	832	ST-828 complex	T86I	tet(O)	NF	A2075G	ERR10372325

C073	Campylobacter coli	2018	3016	ST-828 complex	T86I+D90N	tet(O)	NF	A2075G	ERR6388529
C074	Campylobacter coli	2018	10839	ST-828 complex	T86I	tet(O)	NF	A2075G	ERR10372446
C075	Campylobacter coli	2018	832	ST-828 complex	T86I	tet(O)	NF	A2075G	ERR10372381
C076	Campylobacter coli	2018	832	ST-828 complex	T86I	tet(O)	NF	A2075G	ERR10372298
C077	Campylobacter coli	2018	832	ST-828 complex	T86I	tet(O)	NF	A2075G	ERR10372448
C078	Campylobacter coli	2018	832	ST-828 complex	T86I	tet(O)	NF	A2075G	ERR10372317
C079	Campylobacter coli	2018	10838	ST-1150 complex	T86I	tet(W)	NF	A2075G	ERR10372450
C080	Campylobacter coli	2018	3016	ST-828 complex	T86I	tet(O)	NF	A2075G	ERR10372356
C081	Campylobacter coli	2018	10832	NA	T86I	tet(O)	NF	A2075G	ERR10372492
C082	Campylobacter coli	2018	832	ST-828 complex	T86I	tet(O)	NF	A2075G	ERR10372404
C083	Campylobacter coli	2018	832	ST-828 complex	T86I	tet(O)	NF	A2075G	ERR10372302
C084	Campylobacter coli	2018	832	ST-828 complex	T86I	tet(O)	NF	A2075G	ERR10372407
C085	Campylobacter coli	2018	10833	NA	T86I	tet(O)	NF	A2075G	ERR10372373
C113	Campylobacter coli	2019	872	ST-828 complex	T86I	tet(O)	aadE-Cc	A2075G	ERR10372465
C114	Campylobacter coli	2019	854	ST-828 complex	T86I	tet(O)	aadE-Cc	A2075G	ERR10372376
C115	Campylobacter coli	2019	854	ST-828 complex	T86I	tet(O)	aadE-Cc	A2075G	ERR10372360
C116	Campylobacter coli	2019	832	ST-828 complex	T86I	tet(O)	NF	A2075G	ERR10372316
C117	Campylobacter coli	2019	860	ST-828 complex	T86I	tet(O)	NF	A2075G	ERR10372428
C118	Campylobacter coli	2019	832	ST-828 complex	T86I	tet(O)	NF	A2075G	ERR10372358
C119	Campylobacter coli	2019	825	ST-828 complex	T86I	tet(O)	aadE-Cc	A2075G	ERR10372431
C120	Campylobacter coli	2019	832	ST-828 complex	T86I	tet(O)	ant(6)-Ia	A2075G	ERR10372494
C121	Campylobacter coli	2019	3016	ST-828 complex	T86I	tet(O)	NF	A2075G	ERR10372418
C122	Campylobacter coli	2019	11397	ST-828 complex	T86I	tet(O)	aadE-Cc	A2075G	ERR10372365
C123	Campylobacter coli	2019	8321	ST-828 complex	T86I	tet(O)	NF	A2075G	ERR10372498
C124	Campylobacter coli	2019	3016	ST-828 complex	T86I	tet(O)	NF	A2075G	ERR10372396
C125	Campylobacter coli	2019	832	ST-828 complex	T86I	tet(O)	NF	A2075G	ERR10372345
C126	Campylobacter coli	2019	9987	ST-828 complex	T86I	tet(O)	aadE-Cc	A2075G	ERR10372301
C127	Campylobacter coli	2019	9987	ST-828 complex	T86I	tet(O)	aadE-Cc	A2075G	ERR10372488
C128	Campylobacter coli	2019	832	ST-828 complex	T86I	tet(O)	NF	A2075G	ERR10372382
C132	Campylobacter coli	2020	5659	ST-828 complex	T86I	tet(O/32/O)	NF	NF	ERR10372478

C133	Campylobacter coli	2020	825	ST-828 complex	T86I	tet(O)	aadE-Cc	NF	ERR10372399
C134	Campylobacter coli	2020	832	ST-828 complex	T86I	tet(O)	NF	NF	ERR10372432
C135	Campylobacter coli	2020	825	ST-828 complex	T86I	tet(O)	aadE-Cc	A2075G	ERR10372426
C136	Campylobacter coli	2020	11414	NA	NF	NF	NF	NF	ERR10372462
C137	Campylobacter coli	2020	1628	ST-828 complex	T86I	NF	NF	NF	ERR10372463
C138	Campylobacter jejuni	2020	11476	ST-353 complex	T86I	tet(O/32/O)	ant(6)-Ia	NF	ERR10372664
C139	Campylobacter jejuni	2020	904	ST-607 complex	T86I	tet(O)	NF	NF	ERR10372597
C140	Campylobacter jejuni	2020	49	ST-49 complex	T86I	NF	NF	NF	ERR10372602
C141	Campylobacter jejuni	2020	1701	ST-45 complex	NF	NF	NF	NF	ERR10372625
C142	Campylobacter jejuni	2020	8579	ST-464 complex	T86I	tet(O/32/O)	NF	NF	ERR10372659
C143	Campylobacter coli	2020	11469	ST-828 complex	T86I	tet(O)	aadE-Cc	NF	ERR10372421
C144	Campylobacter jejuni	2020	22	ST-22 complex	NF	NF	NF	NF	ERR10372783
C145	Campylobacter coli	2020	11467	ST-828 complex	T86I	tet(O/32/O)	NF	NF	ERR10372367
C146	Campylobacter coli	2020	832	ST-828 complex	T86I	tet(O)	NF	A2075G	ERR10372312
C147	Campylobacter coli	2020	10042	ST-828 complex	T86I	tet(O/32/O)	NF	NF	ERR10372438
C148	Campylobacter jejuni	2020	49	ST-49 complex	T86I	NF	NF	NF	ERR10372521
C149	Campylobacter coli	2020	825	ST-828 complex	NF	tet(O)	aadE-Cc	NF	ERR10372332
C151	Campylobacter coli	2020	872	ST-828 complex	T86I	tet(O)	aadE-Cc	A2075G	ERR10372322
C152	Campylobacter coli	2020	3016	ST-828 complex	T86I	tet(O)	NF	A2075G	ERR10372366
C153	Campylobacter coli	2020	827	ST-828 complex	NF	NF	aadE-Cc	NF	ERR10372435
C154	Campylobacter coli	2020	832	ST-828 complex	T86I	tet(O)	NF	A2075G	ERR10372490
C155	Campylobacter coli	2020	855	ST-828 complex	T86I	tet(O)	NF	NF	ERR10372456
C156	Campylobacter coli	2020	1628	ST-828 complex	T86I	NF	NF	NF	ERR10372342
C157	Campylobacter coli	2020	828	ST-828 complex	T86I	tet(O)	aadE-Cc	A2075G	ERR10372337
C158	Campylobacter coli	2020	825	ST-828 complex	NF	tet(O)	aadE-Cc	NF	ERR10372501
C159	Campylobacter coli	2020	11399	NA	T86I	tet(O)	NF	A2075G	ERR10372379
C160	Campylobacter coli	2020	1628	ST-828 complex	T86I	NF	NF	NF	ERR10372483
C180	Campylobacter coli	2021	9987	ST-828 complex	T86I	tet(O)	aadE-Cc + ant(6)-Ia	A2075G	ERR10372362
C181	Campylobacter coli	2021	6510	ST-828 complex	T86I	tet(O)	aadE-Cc	NF	ERR10372387
C182	Campylobacter coli	2021	11397	ST-828 complex	T86I	tet(O)	aadE-Cc	A2075G	ERR10372394

C183	Campylobacter coli	2021	1595	ST-828 complex	T86I	tet(O)	ant(6)-Ia	NF	ERR10372338
C184	Campylobacter coli	2021	1595	ST-828 complex	T86I	tet(O)	ant(6)-Ia	NF	ERR10372328
C185	Campylobacter coli	2021	11397	ST-828 complex	T86I	tet(O)	aadE-Cc	A2075G	ERR10372392
C186	Campylobacter coli	2021	11397	ST-828 complex	T86I	tet(O)	aadE-Cc	A2075G	ERR10372386
C187	Campylobacter coli	2021	10042	ST-828 complex	T86I	tet(O/32/O)	NF	A2075G	ERR10372476
C188	Campylobacter coli	2021	8195	ST-828 complex	T86I	tet(O/32/O)	ant(6)-Ia	NF	ERR10372388
C189	Campylobacter coli	2021	10042	ST-828 complex	T86I	tet(O/32/O)	NF	A2075G	ERR10372486
C190	Campylobacter coli	2021	10042	ST-828 complex	T86I	tet(O/32/O)	ant(6)-Ia	A2075G	ERR10372441
C191	Campylobacter coli	2021	2699	ST-828 complex	T86I	tet(O)	aadE-Cc	A2075G	ERR10372310
C192	Campylobacter coli	2020	3017	ST-828 complex	T86I	tet(O)	NF	NF	ERR10372493
C193	Campylobacter coli	2021	828	ST-828 complex	T86I	tet(O)	aadE-Cc	A2075G	ERR10372477
C194	Campylobacter coli	2021	8518	NA	T86I	tet(O)	NF	NF	ERR10372406
C195	Campylobacter coli	2021	10042	ST-828 complex	T86I	tet(O/32/O)	NF	NF	ERR10372452
C196	Campylobacter coli	2021	9987	ST-828 complex	T86I	tet(O)	aadE-Cc	A2075G	ERR10372499
C197	Campylobacter coli	2021	9987	ST-828 complex	T86I	tet(O)	aadE-Cc	A2075G	ERR10372343
C198	Campylobacter coli	2021	11397	ST-828 complex	T86I	tet(O)	aadE-Cc	A2075G	ERR10372311
C199	Campylobacter coli	2021	855	ST-828 complex	T86I	tet(O)	NF	NF	ERR10372350
C200	Campylobacter jejuni	2021	572	ST-206 complex	T86I	tet(O/32/O)	ant(6)-Ia	NF	ERR10372520
C201	Campylobacter jejuni	2021	6461	ST-353 complex	T86I	tet(O/32/O)	ant(6)-Ia	NF	ERR10372706
C202	Campylobacter jejuni	2021	50	ST-21 complex	T86I	NF	NF	NF	ERR10372548
C203	Campylobacter jejuni	2021	824	ST-257 complex	T86I	NF	NF	NF	ERR10372721
C204	Campylobacter jejuni	2021	607	ST-607 complex	T86I	tet(O)	NF	NF	ERR10372510
C205	Campylobacter jejuni	2021	122	ST-206 complex	T86I	NF	NF	NF	ERR10372657
C206	Campylobacter jejuni	2021	607	ST-607 complex	T86I	tet(O)	NF	NF	ERR10372661
C207	Campylobacter jejuni	2021	10846	ST-353 complex	T86I	tet(O/32/O)	NF	NF	ERR10372747
C208	Campylobacter jejuni	2021	10622	NA	T86I	tet(O)	NF	A2075G	ERR10372643
C209	Campylobacter jejuni	2021	354	ST-354 complex	T86I	tet(O)	NF	NF	ERR10372591
C210	Campylobacter jejuni	2021	6522	ST-443 complex	T86I	NF	NF	NF	ERR10372513
C211	Campylobacter jejuni	2021	354	ST-354 complex	T86I	tet(O)	NF	NF	ERR10372559
C212	Campylobacter jejuni	2021	6522	ST-443 complex	T86I	NF	NF	NF	ERR10372680

C213	Campylobacter jejuni	2021	6461	ST-353 complex	T86I	tet(O/32/O)	ant(6)-Ia	NF	ERR10372748
C214	Campylobacter jejuni	2021	10846	ST-353 complex	T86I	tet(O/32/O)	NF	NF	ERR10372691
C215	Campylobacter jejuni	2021	2180	ST-658 complex	T86I	tet(O)	NF	NF	ERR10372689
C216	Campylobacter jejuni	2021	49	ST-49 complex	T86I	NF	NF	NF	ERR10372628
C217	Campylobacter jejuni	2021	50	ST-21 complex	T86I	tet(O)	NF	NF	ERR10372580
C218	Campylobacter jejuni	2021	7517	ST-443 complex	T86I	NF	NF	NF	ERR10372654
C219	Campylobacter jejuni	2021	1044	ST-658 complex	T86I	NF	NF	NF	ERR10372618
C220	Campylobacter jejuni	2021	2258	NA	T86I	NF	NF	NF	ERR10372761
C221	Campylobacter jejuni	2021	8579	ST-464 complex	T86I	tet(O/32/O)	NF	NF	ERR10372694
C222	Campylobacter jejuni	2021	52	ST-52 complex	T86I	NF	NF	NF	ERR10372718
C223	Campylobacter jejuni	2021	10846	ST-353 complex	T86I	tet(O/32/O)	NF	NF	ERR10372633
C224	Campylobacter jejuni	2021	51	ST-443 complex	T86I	tet(O/32/O)	NF	NF	ERR10372777
C225	Campylobacter jejuni	2021	50	ST-21 complex	T86I	NF	NF	NF	ERR10372624
C226	Campylobacter jejuni	2021	8332	ST-464 complex	T86I	tet(O/32/O)	NF	NF	ERR10372592
C227	Campylobacter jejuni	2021	1044	ST-658 complex	T86I	NF	NF	NF	ERR10372780
C228	Campylobacter jejuni	2021	2274	NA	T86I	tet(O)	NF	NF	ERR10372698
C229	Campylobacter jejuni	2021	9887	ST-353 complex	T86I	tet(O)	NF	NF	ERR10372598
C230	Campylobacter jejuni	2021	7114	NA	T86I	tet(O)	NF	NF	ERR10372699
C231	Campylobacter jejuni	2021	531	NA	T86I	tet(O)	NF	NF	ERR10372712
C232	Campylobacter jejuni	2021	9887	ST-353 complex	T86I	tet(O)	NF	NF	ERR10372656
C233	Campylobacter coli	2021	11473	NA	T86I	tet(O)	NF	A2075G	ERR10372459
C234	Campylobacter jejuni	2021	531	NA	T86I	tet(O)	NF	NF	ERR10372566
C235	Campylobacter jejuni	2021	356	ST-353 complex	T86I	tet(O)	NF	NF	ERR10372655
C236	Campylobacter coli	2021	11470	NA	T86I	tet(W)	NF	A2075G	ERR10372453
C237	Campylobacter jejuni	2021	607	ST-607 complex	T86I	tet(O)	NF	NF	ERR10372626
C238	Campylobacter coli	2018	832	ST-828 complex	T86I	tet(O)	NF	A2075G	ERR10372361
C239	Campylobacter coli	2017	8104	ST-828 complex	T86I	tet(O)	NF	A2075G	ERR10372479
C244	Campylobacter coli	2019	3016	ST-828 complex	T86I	tet(O)	NF	NF	ERR10372405
C245	Campylobacter coli	2019	2177	ST-828 complex	T86I+D90N	tet(O)	NF	A2075G	ERR10372434
C246	Campylobacter coli	2019	832	ST-828 complex	T86I	tet(O)	NF	A2075G	ERR10372304

C247	Campylobacter coli	2019	832	ST-828 complex	T86I	tet(O)	NF	A2075G	ERR10372329
C248	Campylobacter coli	2019	832	ST-828 complex	T86I	tet(O)	NF	A2075G	ERR10372491
C249	Campylobacter coli	2021	9987	ST-828 complex	T86I	tet(O)	aadE-Cc	A2075G	ERR10372353
C001	Campylobacter jejuni	2016	50	ST-21 complex	T86I	NF	NF	NF	ERR10372586
C002	Campylobacter jejuni	2016	50	ST-21 complex	T86I	tet(O)	NF	NF	ERR10372741
C003	Campylobacter jejuni	2016	11425	ST-48 complex	T86I	NF	NF	NF	ERR10372732
C004	Campylobacter jejuni	2016	2254	ST-257 complex	T86I	tet(O)	NF	NF	ERR10372525
C005	Campylobacter jejuni	2016	122	ST-206 complex	T86I	tet(O/32/O)	NF	NF	ERR10372678
C006	Campylobacter jejuni	2016	990	ST-257 complex	T86I	tet(O/32/O)	NF	NF	ERR10372601
C007	Campylobacter jejuni	2016	990	ST-257 complex	T86I	tet(O/32/O)	NF	NF	ERR10372692
C008	Campylobacter jejuni	2016	11427	ST-607 complex	T86I	tet(O)	NF	NF	ERR10372770
C009	Campylobacter jejuni	2016	122	ST-206 complex	T86I	tet(O/32/O)	NF	NF	ERR10372526
C010	Campylobacter jejuni	2016	11428	ST-21 complex	T86I	tet(O/32/O)	NF	NF	ERR10372634
C011	Campylobacter jejuni	2016	772	ST-443 complex	T86I	tet(O)	NF	NF	ERR10372541
C012	Campylobacter jejuni	2016	49	ST-49 complex	T86I	NF	NF	NF	ERR10372700
C013	Campylobacter jejuni	2016	607	ST-607 complex	T86I	tet(O)	NF	NF	ERR10372561
C014	Campylobacter jejuni	2016	904	ST-607 complex	T86I	tet(O)	NF	A2074N	ERR6394672
C015	Campylobacter jejuni	2016	2036	ST-353 complex	T86I	tet(O)	NF	A2075G	ERR10372511
C016	Campylobacter jejuni	2016	356	ST-353 complex	T86I	tet(O)	NF	NF	ERR10372784
C017	Campylobacter jejuni	2016	10622	NA	T86I	tet(O)	NF	A2075G	ERR10372740
C018	Campylobacter jejuni	2017	21	ST-21 complex	T86I	tet(O)	NF	NF	ERR10372576
C019	Campylobacter jejuni	2017	44	ST-21 complex	T86I	tet(O)	NF	NF	ERR10372533
C020	Campylobacter jejuni	2017	990	ST-257 complex	T86I	tet(O/32/O)	NF	NF	ERR10372505
C021	Campylobacter jejuni	2017	904	ST-607 complex	T86I	tet(O)	NF	NF	ERR10372767
C022	Campylobacter jejuni	2017	354	ST-354 complex	NF	tet(O)	NF	NF	ERR10372539
C023	Campylobacter jejuni	2017	1232	ST-353 complex	T86I	tet(O)	NF	NF	ERR10372792
C024	Campylobacter jejuni	2017	47	ST-21 complex	T86I	tet(O/32/O)	NF	NF	ERR10372729
C025	Campylobacter jejuni	2017	1232	ST-353 complex	T86I	tet(O)	NF	NF	ERR10372579
C026	Campylobacter jejuni	2017	607	ST-607 complex	T86I	tet(O)	NF	NF	ERR10372507
C027	Campylobacter jejuni	2017	990	ST-257 complex	T86I	tet(O/32/O)	NF	NF	ERR10372751

C028	Campylobacter jejuni	2017	148	ST-21 complex	T86I	NF	NF	NF	ERR10372762
C029	Campylobacter jejuni	2017	400	ST-353 complex	T86I	tet(O/32/O)	NF	NF	ERR10372519
C030	Campylobacter jejuni	2017	50	ST-21 complex	T86I	NF	NF	NF	ERR10372754
C031	Campylobacter jejuni	2017	11430	ST-21 complex	NF	NF	NF	NF	ERR10372616
C032	Campylobacter jejuni	2017	11434	ST-21 complex	T86I	NF	NF	NF	ERR10372572
C033	Campylobacter jejuni	2017	47	ST-21 complex	T86I	tet(O/32/O)	NF	NF	ERR10372515
C034	Campylobacter jejuni	2017	122	ST-206 complex	T86I	tet(O/32/O)	NF	NF	ERR10372516
C035	Campylobacter jejuni	2017	11436	ST-21 complex	T86I	NF	NF	NF	ERR10372667
C036	Campylobacter jejuni	2017	6522	ST-443 complex	T86I	NF	NF	NF	ERR10372757
C037	Campylobacter jejuni	2017	47	ST-21 complex	T86I	tet(O/32/O)	NF	NF	ERR10372518
C038	Campylobacter jejuni	2017	443	ST-443 complex	T86I	tet(O/32/O)	NF	NF	ERR10372705
C039	Campylobacter jejuni	2017	52	ST-52 complex	T86I	NF	NF	NF	ERR10372571
C040	Campylobacter jejuni	2017	443	ST-443 complex	T86I	tet(O/32/O)	NF	NF	ERR10372635
C041	Campylobacter jejuni	2017	475	ST-48 complex	T86I	NF	NF	NF	ERR10372538
C042	Campylobacter jejuni	2017	572	ST-206 complex	T86I	tet(O)	NF	NF	ERR10372758
C043	Campylobacter jejuni	2017	50	ST-21 complex	T86I	NF	NF	NF	ERR10372703
C044	Campylobacter jejuni	2017	10622	NA	T86I	tet(O)	NF	A2075G	ERR10372743
C045	Campylobacter jejuni	2017	904	ST-607 complex	T86I	tet(O)	NF	A2074N	ERR10372508
C046	Campylobacter jejuni	2017	354	ST-354 complex	T86I	tet(O)	NF	NF	ERR10372503
C047	Campylobacter jejuni	2017	50	ST-21 complex	T86I	tet(O)	NF	A2075G	ERR10372611
C048	Campylobacter jejuni	2018	354	ST-354 complex	T86I	tet(O)	NF	A2075G	ERR10372645
C049	Campylobacter jejuni	2018	990	ST-257 complex	T86I	tet(O/32/O)	NF	NF	ERR10372504
C050	Campylobacter jejuni	2018	354	ST-354 complex	T86I	tet(O)	NF	A2075G	ERR10372776
C051	Campylobacter jejuni	2018	572	ST-206 complex	T86I	tet(O)	NF	NF	ERR10372543
C052	Campylobacter jejuni	2018	8971	ST-22 complex	NF	NF	NF	NF	ERR10372794
C053	Campylobacter jejuni	2018	148	ST-21 complex	T86I	NF	NF	NF	ERR10372716
C054	Campylobacter jejuni	2018	11411	NA	T86I	tet(O)	NF	NF	ERR10372529
C055	Campylobacter jejuni	2018	904	ST-607 complex	T86I	tet(O)	NF	NF	ERR10372547
C056	Campylobacter jejuni	2018	572	ST-206 complex	T86I	tet(O)	NF	NF	ERR10372613
C057	Campylobacter jejuni	2018	52	ST-52 complex	T86I	NF	NF	NF	ERR10372523

C058	Campylobacter jejuni	2018	46	ST-206 complex	T86I	tet(O)	NF	NF	ERR10372679
C059	Campylobacter jejuni	2018	44	ST-21 complex	T86I	tet(O)	NF	NF	ERR10372581
C060	Campylobacter jejuni	2018	607	ST-607 complex	T86I	tet(O)	NF	NF	ERR10372549
C061	Campylobacter jejuni	2018	990	ST-257 complex	T86I	tet(O/32/O)	NF	NF	ERR10372564
C062	Campylobacter jejuni	2018	1232	ST-353 complex	T86I	tet(O)	NF	NF	ERR10372512
C063	Campylobacter jejuni	2018	990	ST-257 complex	T86I	tet(O/32/O)	NF	NF	ERR10372568
C064	Campylobacter jejuni	2018	464	ST-464 complex	T86I	tet(O/32/O)	NF	NF	ERR10372707
C065	Campylobacter jejuni	2018	44	ST-21 complex	T86I	tet(O)	NF	NF	ERR10372524
C066	Campylobacter jejuni	2018	44	ST-21 complex	T86I	tet(O)	NF	NF	ERR10372641
C067	Campylobacter jejuni	2018	9887	ST-353 complex	T86I	tet(O)	NF	NF	ERR10372558
C068	Campylobacter jejuni	2018	572	ST-206 complex	T86I	tet(O)	NF	NF	ERR10372738
C069	Campylobacter jejuni	2018	572	ST-206 complex	T86I	tet(O)	NF	NF	ERR10372514
C070	Campylobacter jejuni	2018	8579	ST-464 complex	T86I	tet(O/32/O)	NF	NF	ERR10372517
C071	Campylobacter jejuni	2018	52	ST-52 complex	T86I	NF	NF	NF	ERR10372670
C072	Campylobacter jejuni	2018	1074	ST-460 complex	T86I	tet(O)	NF	NF	ERR10372584
C073	Campylobacter jejuni	2018	3769	ST-21 complex	T86I	tet(O)	NF	NF	ERR10372506
C074	Campylobacter jejuni	2018	1232	ST-353 complex	T86I	tet(O)	NF	NF	ERR10372717
C075	Campylobacter jejuni	2018	990	ST-257 complex	T86I	tet(O/32/O)	NF	NF	ERR10372725
C076	Campylobacter jejuni	2018	1044	ST-658 complex	T86I	NF	NF	NF	ERR10372771
C077	Campylobacter jejuni	2018	8334	ST-353 complex	T86I	tet(O)	NF	NF	ERR10372787
C078	Campylobacter jejuni	2018	607	ST-607 complex	T86I	tet(O)	NF	NF	ERR10372594
C079	Campylobacter jejuni	2018	572	ST-206 complex	T86I	tet(O)	NF	NF	ERR10372759
C080	Campylobacter jejuni	2018	122	ST-206 complex	T86I	tet(O/32/O)	NF	NF	ERR10372527
C081	Campylobacter jejuni	2018	8579	ST-464 complex	T86I	tet(O/32/O)	NF	NF	ERR10372701
C082	Campylobacter jejuni	2018	122	ST-206 complex	NF	NF	NF	NF	ERR10372567
C083	Campylobacter jejuni	2018	572	ST-206 complex	T86I	tet(O)	NF	NF	ERR10372531
C084	Campylobacter jejuni	2018	11442	ST-257 complex	T86I	tet(O/32/O)	NF	NF	ERR10372647
C085	Campylobacter jejuni	2018	11443	ST-464 complex	T86I	tet(O/32/O)	NF	NF	ERR10372736
C086	Campylobacter jejuni	2018	990	ST-257 complex	T86I	tet(O/32/O)	NF	NF	ERR10372789
C087	Campylobacter jejuni	2018	10622	NA	T86I	tet(O)	NF	A2075G	ERR10372674

C090	Campylobacter jejuni	2019	607	ST-607 complex	T86I	tet(O)	NF	NF	ERR10372781
C091	Campylobacter jejuni	2019	1707	ST-607 complex	T86I	tet(O/32/O)	NF	NF	ERR10372606
C092	Campylobacter jejuni	2019	122	ST-206 complex	T86I	NF	NF	NF	ERR10372681
C093	Campylobacter jejuni	2019	534	NA	NF	NF	NF	NF	ERR10372702
C094	Campylobacter jejuni	2019	6532	ST-42 complex	T86I	tet(O/32/O)	ant(6)-Ia	NF	ERR10372765
C095	Campylobacter jejuni	2019	53	ST-21 complex	T86I	tet(O)	NF	NF	ERR10372555
C096	Campylobacter jejuni	2019	44	ST-21 complex	T86I	tet(O)	NF	NF	ERR10372565
C097	Campylobacter jejuni	2019	990	ST-257 complex	T86I	tet(O/32/O)	NF	NF	ERR10372676
C098	Campylobacter jejuni	2019	46	ST-206 complex	T86I	NF	NF	NF	ERR10372537
C099	Campylobacter jejuni	2019	11446	ST-21 complex	T86I	NF	NF	NF	ERR10372693
C100	Campylobacter jejuni	2019	3769	ST-21 complex	T86I	tet(O)	NF	NF	ERR10372713
C101	Campylobacter jejuni	2019	49	ST-49 complex	T86I	NF	NF	NF	ERR10372619
C102	Campylobacter jejuni	2019	49	ST-49 complex	T86I	NF	NF	NF	ERR10372788
C103	Campylobacter jejuni	2019	607	ST-607 complex	T86I	tet(O)	NF	NF	ERR10372583
C104	Campylobacter jejuni	2019	2180	ST-658 complex	T86I	tet(O)	NF	NF	ERR10372763
C105	Campylobacter jejuni	2019	859	ST-443 complex	T86I	tet(O)	NF	NF	ERR10372662
C106	Campylobacter jejuni	2019	2153	ST-581 complex	T86I	tet(O)	NF	NF	ERR10372756
C107	Campylobacter jejuni	2019	1044	ST-658 complex	T86I	NF	NF	NF	ERR10372769
C108	Campylobacter jejuni	2019	354	ST-354 complex	T86I	tet(O)	NF	NF	ERR10372560
C109	Campylobacter jejuni	2019	2036	ST-353 complex	T86I	tet(O)	NF	A2075G	ERR10372612
C110	Campylobacter jejuni	2019	2036	ST-353 complex	T86I	tet(O)	NF	A2075G	ERR10372671
C111	Campylobacter jejuni	2019	607	ST-607 complex	T86I	tet(O)	NF	NF	ERR10372551
C112	Campylobacter jejuni	2019	572	ST-206 complex	T86I	tet(O)	NF	NF	ERR10372631
C113	Campylobacter jejuni	2019	1044	ST-658 complex	NF	NF	NF	NF	ERR10372775
C114	Campylobacter jejuni	2019	572	ST-206 complex	T86I	tet(O)	NF	NF	ERR10372632
C115	Campylobacter jejuni	2019	3769	ST-21 complex	T86I	tet(O)	NF	NF	ERR10372528
C116	Campylobacter jejuni	2019	122	ST-206 complex	T86I	NF	NF	NF	ERR10372697
C117	Campylobacter jejuni	2019	122	ST-206 complex	NF	NF	NF	NF	ERR10372669
C118	Campylobacter jejuni	2019	534	NA	NF	NF	NF	NF	ERR10372760
C119	Campylobacter jejuni	2019	52	ST-52 complex	T86I	NF	NF	NF	ERR10372687

C120	Campylobacter jejuni	2019	572	ST-206 complex	T86I	tet(O)	NF	NF	ERR10372556
C131	Campylobacter jejuni	2019	10622	NA	T86I	tet(O)	NF	A2075G	ERR10372730
C137	Campylobacter jejuni	2020	882	NA	T86I	tet(O)	NF	NF	ERR10372715
C138	Campylobacter jejuni	2020	50	ST-21 complex	T86I	NF	NF	NF	ERR10372535
C139	Campylobacter jejuni	2020	8579	ST-464 complex	T86I	tet(O/32/O)	NF	NF	ERR10372621
C140	Campylobacter jejuni	2020	50	ST-21 complex	T86I	NF	NF	NF	ERR10372726
C141	Campylobacter jejuni	2020	8334	ST-353 complex	T86I	tet(O)	NF	NF	ERR10372653
C142	Campylobacter jejuni	2020	7517	ST-443 complex	T86I	NF	NF	NF	ERR10372711
C143	Campylobacter jejuni	2020	52	ST-52 complex	T86I	NF	NF	NF	ERR10372562
C144	Campylobacter jejuni	2020	50	ST-21 complex	T86I	NF	NF	NF	ERR10372596
C145	Campylobacter jejuni	2020	990	ST-257 complex	T86I	tet(O/32/O)	NF	NF	ERR10372552
C146	Campylobacter jejuni	2020	50	ST-21 complex	T86I	NF	NF	NF	ERR10372720
C147	Campylobacter jejuni	2020	122	ST-206 complex	T86I	tet(O/32/O)	NF	NF	ERR10372778
C148	Campylobacter jejuni	2020	122	ST-206 complex	T86I	tet(O/32/O)	NF	NF	ERR10372724
C149	Campylobacter jejuni	2020	403	ST-403 complex	NF	NF	NF	NF	ERR10372663
C150	Campylobacter jejuni	2020	354	ST-354 complex	T86I	tet(O)	NF	NF	ERR10372639
C151	Campylobacter jejuni	2020	49	ST-49 complex	T86I	NF	NF	NF	ERR10372630
C152	Campylobacter jejuni	2020	6461	ST-353 complex	T86I	tet(O/32/O)	ant(6)-Ia	NF	ERR5922431
C153	Campylobacter jejuni	2020	354	ST-354 complex	T86I	tet(O)	NF	NF	ERR10372786
C154	Campylobacter jejuni	2020	354	ST-354 complex	T86I	tet(O)	NF	NF	ERR10372610
C155	Campylobacter jejuni	2020	22	ST-22 complex	T86I	tet(O)	NF	NF	ERR5922432
C156	Campylobacter jejuni	2020	122	ST-206 complex	NF	NF	NF	NF	ERR10372623
C157	Campylobacter jejuni	2020	122	ST-206 complex	T86I	NF	NF	NF	ERR10372672
C158	Campylobacter jejuni	2020	356	ST-353 complex	T86I	tet(O)	NF	NF	ERR10372587
C159	Campylobacter jejuni	2020	1962	NA	T86I	NF	NF	NF	ERR10372793
C160	Campylobacter jejuni	2020	824	ST-257 complex	NF	NF	NF	NF	ERR10372709
C161	Campylobacter jejuni	2020	583	ST-45 complex	T86I	NF	NF	NF	ERR10372608
C162	Campylobacter jejuni	2020	22	ST-22 complex	NF	NF	NF	NF	ERR5922433
C163	Campylobacter jejuni	2020	583	ST-45 complex	NF	NF	NF	NF	ERR10372627
C164	Campylobacter jejuni	2020	49	ST-49 complex	T86I	NF	NF	NF	ERR10372590

C165	Campylobacter jejuni	2020	50	ST-21 complex	T86I	NF	NF	NF	ERR10372668
C166	Campylobacter jejuni	2020	2180	ST-658 complex	T86I	NF	NF	NF	ERR10372666
C167	Campylobacter jejuni	2020	45	ST-45 complex	NF	tet(O)	NF	NF	ERR10372563
C168	Campylobacter jejuni	2020	10622	NA	T86I	tet(O)	NF	A2075G	ERR10372708
C169	Campylobacter jejuni	2020	354	ST-354 complex	T86I	tet(O)	NF	NF	ERR10372554
C170	Campylobacter jejuni	2020	2153	ST-581 complex	T86I	tet(O)	NF	NF	ERR10372739
C171	Campylobacter jejuni	2020	464	ST-464 complex	T86I	tet(O/32/O)	NF	NF	ERR10372675
C172	Campylobacter jejuni	2020	50	ST-21 complex	NF	tet(O/32/O)	NF	NF	ERR10372768
C173	Campylobacter jejuni	2020	6522	ST-443 complex	T86I	NF	NF	NF	ERR10372677
C174	Campylobacter jejuni	2020	8579	ST-464 complex	T86I	tet(O/32/O)	NF	NF	ERR10372683
C175	Campylobacter jejuni	2020	22	ST-22 complex	NF	NF	NF	NF	ERR5922442
C176	Campylobacter jejuni	2020	5	ST-353 complex	T86I	tet(O)	NF	NF	ERR10372578
C177	Campylobacter jejuni	2020	572	ST-206 complex	T86I	tet(O/32/O)	ant(6)-Ia	NF	ERR10372746
C178	Campylobacter jejuni	2020	122	ST-206 complex	T86I	tet(O/32/O)	NF	NF	ERR10372648
C179	Campylobacter jejuni	2020	122	ST-206 complex	T86I	tet(O/32/O)	NF	NF	ERR10372686
C180	Campylobacter jejuni	2020	50	ST-21 complex	T86I	tet(O/32/O)	NF	NF	ERR10372753
C210	Campylobacter jejuni	2020	904	ST-607 complex	T86I	tet(O)	NF	NF	ERR10372734
C211	Campylobacter jejuni	2020	122	ST-206 complex	NF	NF	NF	NF	ERR10372644
C213	Campylobacter jejuni	2021	508	ST-508 complex	NF	NF	NF	NF	ERR10372682
C214	Campylobacter jejuni	2021	354	ST-354 complex	T86I	tet(O)	NF	NF	ERR10372569
C215	Campylobacter jejuni	2021	6522	ST-443 complex	T86I	NF	NF	NF	ERR10372534
C216	Campylobacter jejuni	2021	1044	ST-658 complex	T86I	NF	NF	NF	ERR10372614
C217	Campylobacter jejuni	2021	1232	ST-353 complex	T86I	tet(O)	NF	NF	ERR10372585
C218	Campylobacter jejuni	2021	990	ST-257 complex	T86I	tet(O/32/O)	NF	NF	ERR10372636
C219	Campylobacter jejuni	2021	3769	ST-21 complex	T86I	tet(O)	NF	NF	ERR10372609
C220	Campylobacter jejuni	2021	7355	ST-353 complex	T86I	tet(O/32/O)	ant(6)-Ia	NF	ERR10372772
C221	Campylobacter jejuni	2021	8332	ST-464 complex	T86I	tet(O/32/O)	NF	NF	ERR10372750
C222	Campylobacter jejuni	2021	11448	ST-443 complex	T86I	tet(O/32/O)	NF	NF	ERR10372577
C223	Campylobacter jejuni	2021	534	NA	NF	NF	NF	NF	ERR10372544
C224	Campylobacter jejuni	2021	122	ST-206 complex	NF	NF	NF	NF	ERR10372575

C225	Campylobacter jejuni	2021	6532	ST-42 complex	T86I	tet(O/32/O)	ant(6)-Ia	NF	ERR10372595
C226	Campylobacter jejuni	2021	2153	ST-581 complex	T86I	tet(O)	NF	NF	ERR10372574
C227	Campylobacter jejuni	2021	11415	ST-353 complex	T86I	tet(O/32/O)	NF	NF	ERR10372774
C228	Campylobacter jejuni	2021	531	NA	T86I	tet(O)	NF	NF	ERR10372522
C229	Campylobacter jejuni	2021	50	ST-21 complex	T86I	NF	NF	NF	ERR10372791
C230	Campylobacter jejuni	2021	607	ST-607 complex	T86I	tet(O)	NF	NF	ERR10372688
C231	Campylobacter jejuni	2021	50	ST-21 complex	T86I	NF	NF	NF	ERR10372755
C232	Campylobacter jejuni	2021	49	ST-49 complex	T86I	NF	NF	NF	ERR10372573
C233	Campylobacter jejuni	2021	918	ST-48 complex	NF	NF	NF	NF	ERR10372540
C234	Campylobacter jejuni	2021	10846	ST-353 complex	T86I	tet(O/32/O)	NF	NF	ERR10372530
C235	Campylobacter jejuni	2021	1759	NA	T86I	tet(O/32/O)	NF	NF	ERR10372735
C236	Campylobacter jejuni	2021	356	ST-353 complex	T86I	tet(O)	NF	NF	ERR10372557
C237	Campylobacter coli	2021	11397	ST-828 complex	T86I	tet(O)	aadE-Cc	A2075G	ERR10372489
C238	Campylobacter jejuni	2021	607	ST-607 complex	T86I	NF	NF	NF	ERR10372696
C239	Campylobacter jejuni	2021	10622	NA	T86I	tet(O)	NF	A2075G	ERR10372620
C240	Campylobacter jejuni	2021	122	ST-206 complex	T86I	tet(O/32/O)	NF	NF	ERR10372737
C241	Campylobacter jejuni	2021	19	ST-21 complex	NF	tet(O)	NF	NF	ERR10372690
C242	Campylobacter jejuni	2021	10622	NA	T86I	tet(O)	NF	A2075G	ERR10372603
C243	Campylobacter jejuni	2018	1707	ST-607 complex	T86I	tet(O/32/O)	NF	NF	ERR10372723
C244	Campylobacter jejuni	2021	10622	NA	T86I	tet(O)	NF	A2075G	ERR10372638
C245	Campylobacter jejuni	2021	657	ST-658 complex	NF	NF	NF	NF	ERR10372785
C246	Campylobacter jejuni	2021	917	ST-21 complex	NF	NF	NF	NF	ERR10372582
C247	Campylobacter jejuni	2021	3769	ST-21 complex	T86I	tet(O)	NF	NF	ERR10372589
C248	Campylobacter jejuni	2021	8332	ST-464 complex	T86I	tet(O/32/O)	NF	NF	ERR10372546
C249	Campylobacter jejuni	2018	859	ST-443 complex	T86I	tet(O)	NF	A2075G	ERR10372509
